# Saprotrophic fungal diversity predicts ectomycorrhizal fungal diversity along the timberline in the framework of island biogeography theory

**DOI:** 10.1038/s43705-021-00015-1

**Published:** 2021-05-18

**Authors:** Teng Yang, Leho Tedersoo, Xiao Fu, Chang Zhao, Xu Liu, Guifeng Gao, Liang Cheng, Jonathan M. Adams, Haiyan Chu

**Affiliations:** 1 State Key Laboratory of Soil and Sustainable Agriculture, Institute of Soil Science, Chinese Academy of Sciences, Nanjing, China; 2 University of Chinese Academy of Sciences, Beijing, China; 3 Mycology and Microbiology Center, University of Tartu, Tartu, Estonia; 4 College of Science, King Saud University, Riyadh, Saudi Arabia; 5 School of Geography Sciences, Nanjing Normal University, Nanjing, China; 6 School of Geographic and Oceanographic Sciences, Nanjing University, Nanjing, China

**Keywords:** Microbial ecology, Biogeography

## Abstract

Island biogeography theory (IBT) is one of the most fruitful paradigms in macroecology, positing positive species-area and negative species-isolation relationships for the distribution of organisms. Biotic interactions are also crucial for diversity maintenance on islands. In the context of a timberline tree species (*Betula ermanii*) as “virtual island”, we surveyed ectomycorrhizal (EcM) fungal diversity along a 430-m vertical gradient on the top of Changbai Mountain, China, sampling fine roots and neighboring soils of *B. ermanii*. Besides elevation, soil properties and plant functional traits, endophytic and saprotrophic fungal diversity were assessed as candidate predictors to construct integrative models. EcM fungal diversity decreased with increasing elevation, and exhibited positive diversity to diameter at breast height and negative diversity to distance from forest edge relationships in both roots and soils. Integrative models further showed that saprotrophic fungal diversity was the strongest predictor of EcM fungal diversity, directly enhancing EcM fungal diversity in roots and soils. Our study supports IBT as a basic framework to explain EcM fungal diversity. The diversity-begets-diversity hypothesis within the fungal kingdom is more predictive for EcM fungal diversity within the IBT framework, which reveals a tight association between saprotrophic and EcM fungal lineages in the timberline ecosystem.

## Introduction

Ectomycorrhizal (EcM) fungi are an important functional guild amongst the soil biota of temperate and boreal forest soils.^1^ They form mutualistic symbioses with fine roots of plants, facilitating seedling establishment and conferring stress resistance.^2^ External EcM mycelium extracts nutrients from organic materials, such as leaf and root litter by Fenton reaction or Mn-peroxidase activities.^3^ Furthermore, EcM fungi can substantially modify forest tree coexistence.^4,5^ Therefore, EcM fungi play a pivotal role in aboveground production enhancement and belowground nutrient cycling in forest ecosystems. Better understanding of EcM fungal diversity will enable us to estimate the effects of global change on forests.^6^

Altitudinal gradients reflect horizontal distribution of biodiversity at a finer spatial scale.^7^ Altitudinal gradients of EcM fungal diversity have revealed four types of patterns: (1) monotonic decrease;^8^ (2) unimodal;^9^ (3) monotonic increase;^10^ (4) no significant change.^11^ The climate-driven hypothesis^12,13^ is most often used to explain the decreasing biodiversity with increasing elevation. A hydrothermal gradient is the basis of the climate-driven hypothesis, e.g., higher fungal diversity occurs in warm and wet rather than cold or dry conditions.^14,15^ However, multicollinearity among elevation, temperature and precipitation makes it difficult to disentangle the relative effects of elevation and climate on diversity,^8,16^ and the climate-driven hypothesis hardly explains other types of elevation patterns. Soil properties may be alternative or complementary to explain EcM fungal diversity along altitudinal gradients. Soil C/N ratio has been found to correlate with EcM fungal diversity along altitudinal gradients.^9,10^ In addition, plant species identity^17^ and root traits^18^ vary across altitude and may influence EcM fungal diversity along altitudinal gradients. By confining studies to a single tree species, we may avoid the confounding impacts of host species and co-occurring plants on EcM fungal diversity.^19,20^

Island biogeography theory (IBT) is one of the core paradigms of macroecology, positing positive species-area and negative species-isolation relationships for plant and animal distributions on islands.^21^ Species-energy theory embodies higher species diversity with increasing available energy, which was thought as a desirable extension of species-area relationship.^22^ Whether IBT fully applies to microbial biogeography is still on debate.^23^ Diffuse populations of EcM trees, as ‘virtual islands’, may provide natural laboratories to testify the application of IBT to EcM fungi.^24^ In coastal pine forests in California, EcM fungal diversity significantly increased with increasing tree island area (<1 to >10 000 m^2^) that explained nearly 74% of the variation in the formula of species-area relationships.^25^ In the same sites, when considering each tree individual as an independent island, they proposed that distance to mature forests predicted nearly half of the variation in EcM fungal diversity.^26^ In subalpine pine forests in Gaylor Lake Basin, USA, researchers also observed a strong positive species-area and negative species-isolation relationships for EcM fungal diversity; the two relationships cumulatively predicted more than one-third of variation in diversity.^27^ The above cases reflect the high universality of IBT on EcM fungal ecology.

EcM fungi co-occur with pathogenic, endophytic, and saprotrophic fungi in belowground ecosystems of natural forests,^28^ forming complex interaction networks.^29,30^ Saprotrophic fungi contribute to a large proportion of soil heterotrophic respiration and obtain C from organic material, which is quite different from C nutrition of EcM fungi.^3^ Most EcM fungal lineages have evolved independently from saprotrophic fungi (i.e., the evolutionary source effect).^2,31^ This evolutionary source effect is assumed to form a positive diversity relationship between EcM and saprotrophic fungi, particularly in a local isolated relict environment. Recently, a large-scale genome sequencing study revealed a transitional process of EcM fungi from saprotrophy to symbiosis and contrasting, lineage-dependent underlying mechanisms.^32^ In addition, a microcosm experiment has shown that various basidiomycete wood-decay fungi can establish facultative biotrophic relationships with EcM plant roots without causing any disease symptoms,^33^ which indicates the possibility of complementary functioning and co-occurrence between saprotrophic and biotrophic fungi in the root niche. Endophytic fungi are commonly termed as a fungal guild that inhabit plants without causing visible disease symptoms.^34,35^ Some EcM fungal species in Sebacinales and Helotiales may have arisen from endophytic ancestors.^36^ Both EcM and endophytic fungi constitute the dominant symbionts in cold and high-elevation regions.^37,38^ Yet, the relationship between diversities of endophytic and EcM fungi is unclear.

Here, we examined EcM fungal diversity in roots and soils of a single EcM tree species (*Betula* *ermanii*) along a 430-m vertical gradient of Changbai Mountain, China. By using integrative modeling (*sensu*^39^), we assessed the causal mechanisms controlling EcM fungal diversity along the timberline, involving the climate-driven hypothesis, IBT, biotic interactions, and effects of soil properties and root biochemical traits. On the basis of multilevel predictors and different ecological frameworks, we hypothesized that (1) IBT is the primary mechanism to predict variation in EcM fungal diversity in the timberline ecosystem, i.e. EcM fungal diversity increases with increasing tree size (diameter at breast height; DBH) and decreasing distance to forest edge (DFE); (2) biotic interactions are an equally important mechanism to predict EcM fungal diversity, i.e., saprotrophic or endophytic fungal diversity directly drive EcM fungal diversity according to the evolutionary source effect. In that case, there would be more EcM fungal species and lineages whenever there are more saprotrophic or endophytic fungal taxa.

## Materials and methods

### Host and site

*B. ermanii* Chamiss, a deciduous broad-leaved tree species, occurs naturally in open spaces within subalpine and boreal forests in Northeast China, Japan and the Russian Far East.^40–42^ *B. ermanii* is also a typical tree species of the timberline, because it can resist to severe frost, strong wind and low temperature by ecophysiological and ecomorphological flexiblity.^43–45^ In addition, a rich mycobiome of *B. ermanii* has been reported,^16,46,47^ which may facilitate the survival and spread of the host plant.

On the northern slope of Changbai Mountain, Northeast China, *B. ermanii* grows over a broad elevation range from ca. 1700 to 2100 m a.s.l., and forms pure stands along >300 m vertical belt below the timberline.^42,48^ The establishment of Changbai Nature Reserve (CNR) in 1960 gave strict protection to the Erman’s birch (*B. ermanii*) forests.^49^ All our sampling was located within the core region of the CNR. The region has a typical continental temperate monsoon climate, with higher elevations experiencing lower temperature and greater precipitation.^50^ Soils of the Erman’s birch forests are Permi-Gelic Cambosols, whereas the soils beyond the upper and lower limits of *B. ermanii* zone are Permafrost cold Cambosols and Umbri-Gelic Cambosols, respectively. Climate change, particularly rising temperature, threatens the populations of *B. ermanii* and the whole Erman’s birch forest ecosystem in Changbai Mountain.^50,51^

### Field sample collection

**Fig. 1:**
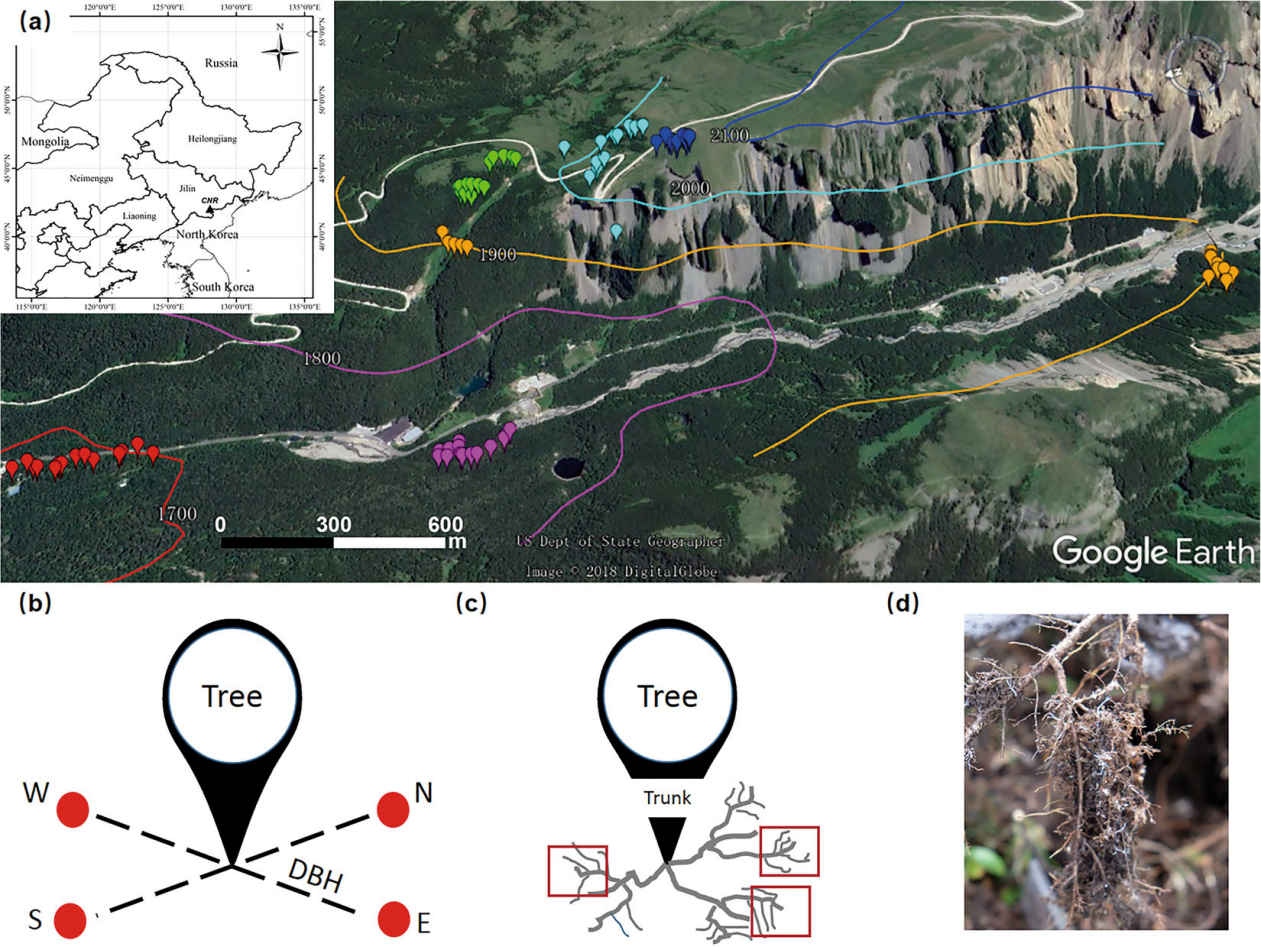
Sampling map and procedures in this study. **a** Sampling map in the core region of CNR: the icons with different colors represent the tree individuals of *B. ermanii*. Contours were fitted in a map of Google Earth. **b** The sampling procedure of neighboring soils: each red point represent one soil core with depth 0–10 cm and diameter 3.5 cm. **c** The sampling procedure of fine roots: each red square (nearly 6 × 8 cm) represent a sub-fine-root system (namely, **d**).

We sampled fine roots and neighboring soils for *B. ermanii* individuals at six elevation-related habitats of *B. ermanii* on 2–8 September, 2018 (Fig. 1a). These six elevation habitats include the upper limit (2069–2116 m), tree islands (1997–2042 m), treeline (1949–1992 m), pure stands (including two sub-sites isolated by over 2 km; 1900–1926 m), ecotone of dark coniferous forests and Erman’s birch forests (1742–1765 m) and lower limit (sparse individuals in coniferous forests; 1688–1706 m), respectively. In each habitat, 14 trees were randomly selected, and all trees were located more than 20 m apart from other sampled trees to ensure independence of each sample. Neighboring soils and fine roots were collected according to the protocols of Yang et al.^28^ and Lankau and Keymer,^52^ respectively. Briefly, with the trunk as center and the DBH as distance from the stem, we collected four soil cores (diameter = 3.5 cm, depth = 10 cm) after removal of litter and mixed them as a single composite soil sample (Fig. 1b). We excavated surface soils near the base of trees and traced roots from the base to terminal fine roots in three directions. The fine roots of three directions were combined as a composite fine root sample, and each raw sub-fine-root section was nearly 6 cm wide and 8 cm long (Fig. 1c, d). All the samples were brought back to the laboratory with ice bags within 8 h. Soil was sieved through a 2-mm mesh and divided into two subsamples: one was stored at 4 °C to determine the soil properties, whereas the other was stored at −40 °C for subsequent DNA extraction. Fine roots were rinsed with sterile water and cut into 1.5-cm segments: one subsample (ca. 80%) was stored at 4 °C to determine root biochemical traits, and the other subsample (ca. 20%) was stored at −40 °C for subsequent DNA extraction. In the field, tree height, canopy diameter, DBH, elevation, slope, latitude, and longitude of each sampled tree were recorded. In total, 84 fine roots and 84 neighboring soils were collected.

### Measurement of soil properties and root traits

We measured 28 soil properties, including soil pH, moisture, conductivity, dissolved organic carbon, dissolved organic nitrogen (DON), ammonium nitrogen, nitrate nitrogen, total carbon, total nitrogen, total phosphate, total potassium, total calcium, total magnesium, total manganese, total iron, total aluminum, available phosphate, available potassium, available calcium, available magnesium, available manganese, available iron, available aluminum, C/N ratio, C/P ratio and the proportions of clay, silt and sand. The measurement methods of soil pH, moisture, ammonium nitrogen, nitrate nitrogen, total carbon, total nitrogen and total content of other elements followed our recent study.^28^ In addition, soil conductivity was determined with a soil to water ratio of 1:5 by conductivity meter (Mettler Toledo FE30, Shanghai, China). Mehlich 3^53^ and three-acid-system (nitric acid, perchloric acid, and hydrofluoric acid) were used to extract the available and total content of elements, respectively. Total and available content of phosphate, potassium, calcium, magnesium, manganese, iron, and aluminum were measured using an ICP Optima 8000 (Perkin-Elmer, Waltham, MA, USA). The proportions of clay, silt, and sand were measured by Laser Particle Sizer LS13320 (Beckman, Brea, CA, USA).

Eighteen root traits, including root total carbon (RTC), root total nitrogen (RTN), root phosphate, root potassium, root calcium, root magnesium, root manganese, root iron, root aluminum, root C/N ratio, root N/P ratio, lignin, cellulose, hemicellulose, soluble sugar, soluble protein, free amino acid (FAA) and free fatty acids (FFA), were also measured. Specifically, RTC and RTN were determined with a carbon–hydrogen–nitrogen (CHN) elemental analyzer (2400 II CHN elemental analyzer; PerkinElmer, Boston, MA, USA). Root phosphate, root potassium, root calcium, root magnesium, root manganese, root iron, and root aluminum were measured in ICP Optima 8000 (Perkin-Elmer, Waltham, MA, USA). Soluble protein was measured by a dye-binding assay.^54^ FAA was analyzed by the amino acid analyzer L-8800 (Hitachi, Tokyo, Japan) with leucine as the standard sample. FFA was determined by NEFA FS kits (Diasys, Holzheim, Garman) and the automatic biochemical analyzer AU680 (Olympus, Tokyo, Japan). The measurement methods of lignin, cellulose, hemicellulose, and soluble sugar followed that of our previous study.^16^

### Calculation of distance to forest edge

The location of each tree individual was determined by latitude and longitude. A high-resolution map (treecover2000) on global forest cover at a spatial resolution of 30 m was used as a base map.^55^ In the map, the areas where forest cover was more than 30% were defined as the “mainland” in the IBT framework and shown as the green grids in ArcGIS (Fig. S1). This standard referred to the proposal of Convention on Climate Change Kyoto.^56^ Then, we calculated the minimum distance of each tree to the neighboring forest edge (i.e., green grids) by using the function *Near* of the *Proximity* tool box in ArcGIS 10.0 (ESRI, Redlands, CA, USA).

### Sequencing and bioinformatics

Soil total DNA was extracted from 0.5 g of soil by using FastDNA^®^ Spin kit for Soil (MP Biomedicals, Solon, Ohio, USA). Total DNA of fine roots was extracted from 0.3 g of plant tissue by using Qiagen Plant DNeasy kits (Qiagen, Hilden, Germany). PCR procedures, including primers (ITS1-F: CTTGGTCATTTAGAGGAAGTAA, ITS2: GCTGCGTTCTTCATCGATGC) and conditions were described in our previous studies.^16,28^ The PCR products of all samples were normalized to equimolar amounts and sequenced on the Illumina MiSeq PE300 platform of the Majorbio Company, Shanghai, China.

We first merged the paired-end reads using FLASH.^57^ QIIME 1.9.0^58^ and Cutadapt 1.9.1^59^ were applied for quality filtering, trimming, and chimera removal. Altogether 8,238,146 sequences passed quality filtering (parameters: minlength = 240; maxambigs = 0; phred quality threshold = 30). ITSx 1.0.11 was used to remove the flanking small ribosomal subunit (SSU) and 5.8 S genes,^60^ leaving the ITS1 region for further analyses. The putative chimeric sequences were removed using a combination of de novo and reference-based chimera checking, with the parameter *–non_chimeras_rentention* = *union* in QIMME.^61^ The remaining sequences were then clustered into operational taxonomic units (OTUs) at 97% similarity threshold by using USEARCH.^62^ Singletons were also removed during the USERCH clustering process. Fungal taxonomy was assigned to each OTU by using the Ribosomal Database Project Classifier with minimum confidence of 0.8.^63^ The UNITE v.8.0 (http://unite.ut.ee) release for QIIME served as a reference database for fungal taxonomy.^64^ The OTU table was then curated with LULU, a post-clustering OTU table curation method, to improve diversity estimates.^65^

After removing non-fungal sequences, the final data set included 7,849,126 fungal sequences covering 6663 OTUs in 168 samples (minimum 4662; maximum 70,779; mean 46,721 sequences per sample). The rarefaction curves of the average observed OTU number are shown in Fig. S2. FUNGuild was used to assign each OTU to a putative functional guild, and the assignments with confidence ranking “possible” were assigned as “unknown” as recommended by the authors.^66^ We further modified the assignment of EcM fungi (the subject in the present study) and their lineages according to.^31^ For some OTUs that were simultaneously assigned to endophytic, saprotrophic, or pathogenic fungi, we considered these as endophytes in roots and saprotrophs in soil samples.

### Statistics

All statistical analyses were conducted in R 3.5.2^67^ and AMOS 21.0 (AMOS IBM, New York, USA). In order to analyze the alpha diversities of soil fungi and the three most dominant guilds (viz., EcM, endophytic and saprotrophic fungi) at the same sequencing depth, the data set was subsampled to 4662 reads with 30 iterations. The mean number of observed OTUs was used to represent the diversities of total fungi, EcM fungi, endophytic fungi, and saprotrophic fungi, as previously implemented in.^15,68^ Numbers of EcM fungal lineages and saprotrophic genera, families, orders, and classes of each sample were also calculated based on the same subsampling.

First, linear and quadratic regression models were used to determine the effect of elevation on diversities of total fungi, EcM fungi, endophytic fungi, and saprotrophic fungi. The model with lowest Akaike’s information criterion (AIC) value was selected. In order to account for spatial effects, linear mixed-effects models (LMMs) were fitted using the lme4 package^69^ to analyze the variation in diversities of total fungi, EcM fungi, endophytic fungi, and saprotrophic fungi along the elevation gradient with latitude and longitude as random factors. Corrected Akaike Information Criterion (AICc) for small data sets was used to identify the best mixed-effects model from linear and quadratic polynomial models. The significance of each LMM was tested by the function *Anova* in the car package.^70^ Marginal (m) and conditional (c) *R*^2^ were calculated by the function *r.squaredGLMM* in the MuMIn package.^71^ Marginal *R*^2^ (*R*^2^_m_) represents the variance explained by fixed effects, whereas conditional *R*^2^ (*R*^2^_c_) represents the variance explained by both fixed and random effects.

Second, to test the application of IBT on EcM fungal diversity, DBH and RTC of *B. ermanii* were chosen as proxies of island area and energy, respectively, whereas DFE was chosen as the proxy of island isolation (i.e., island distance to mainland). Linear regression models were used to assess the species-area, species-energy, and species-isolation relationships. In order to account for spatial effects, LMMs were also used for these independent relationships with latitude and longitude as random factors as described above. Classical power-law function models were used to identify the species-area relationship for EcM fungal diversities in roots and soils using *z*-values in the formula S = CA^z^ ^72^ to compare EcM fungi with macroorganisms in previous studies. Furthermore, ordinary least squares (OLS) multiple regression models were performed to identify the relative contributions of DBH, RTC, and DFE on pattern of EcM fungal diversity when considering other predictive variables. Here, five spatial vectors (PCNM1-5) with significant positive spatial autocorrelation (Fig. S3) were obtained by the principal coordinates of neighbor matrices (PCNM) method,^73^ and added into OLS multiple regression models to consider the possible geographic effect. EcM fungal diversities in roots and soils, 28 soil properties, 18 root traits, elevation, slopes, DBH, tree height, canopy diameter, and DFE were standardized (average = 0 and SD = 1) before the OLS multiple regression analysis. AIC was used to identify the best OLS multiple regression model, as implemented in the MASS package.^74^ Variance inflation factor (VIF) was calculated for each model by the function *vif* in the car package. We used the criterion VIF < 3 to adjust to multicollinearity of predictive variables. The function *forward.sel* in the packfor package^75^ was implemented to estimate the relative contributions of each predictive variable on the variation in EcM fungal diversity. The best OLS multiple regression models in this step are called as OLS multiple regression models #1 to distinguish these from the following models.

Third, to test the possible effect of biotic interactions on EcM fungal diversity, diversities of endophytic and saprotrophic fungi in soils and roots were added into the OLS multiple regression models. These resulted in best OLS multiple regression models #2. The significant differences between OLS multiple regression models #1 and #2 were identified by the function *anova* in the stats package.^67^ In addition, partial least squares regression (PLSR) was performed to identify the effect of biotic interactions (i.e., saprotrophic fungal diversity) on EcM fungal diversity, as implemented in the pls package.^76^ In order to test the evolutionary source effect within the fungal kingdom, PLSR was also used to identify the relationships between EcM fungal lineages and saprotrophic fungal taxa at the genus, family, order, and class levels, respectively.

Finally, we used the integrated model (i.e., structural equation modeling (SEM)) to combine different ecological frameworks, including IBT, biotic interactions, the climate-driven hypothesis and effects of soil properties and root traits, to predict EcM fungal diversity along the timberline. Specifically, (1) we built a SEM theoretical model on EcM fungal diversity (Fig. S4), in which elevation, island isolation, island area, island energy, biotic interactions, and soil properties acted as ‘predictive aspects’. (2) Based on the best fitted models among OLS multiple regression models #1 and #2, we added the corresponding predictive variables to SEM: Our first step was to assign the predictive variables from OLS multiple regression models to each ‘predictive aspects’ in SEM by 1:1 (e.g., DFE as island isolation and DBH as island area). Because there were more than one of soil properties and root traits screened in the best OLS multiple regression models, we then continued to add other predictive variables into SEM stepwise. (3) AIC was used to screen the best SEM model among alternative models. The total, direct and indirect effects of each ‘predictive aspects’ were interpreted by summing the standardized path coefficient (SPC). Alternatively, variation partitioning analysis (VPA) was performed to identify the shared and independent contributions of IBT, biotic interactions, and other predictors (i.e., elevation and soil properties) on EcM fungal diversity by the function *varpart* in the vegan package.^77^

## Results

### Data characteristics

In total, 7,849,126 high-quality sequences from 168 samples (incl. 84 roots and 84 soils) were obtained and clustered into 6663 fungal OTUs. Of these, 3204 OTUs were assigned to three dominant guilds (viz., EcM, endophytic and saprotrophic fungi), which accounted for 75.3% of total sequences (Table S1). In EcM fungi, 1,344,174 sequences (793 OTUs) and 1,952,644 sequences (1015 OTUs) were assigned to 35 and 38 lineages in roots and soils, respectively. The /russula-lactarius (55.0%), /tomentella-thelephora (12.9%) and /piloderma (11.7%) were dominant EcM fungal lineages in roots, whereas /russula-lactarius (43.3%), /tomentella-thelephora (9.7%) and /cortinarius (9.0%) were dominant lineages in soils (Table S2). Based on the rarefied data set, diversity of total fungi, EcM fungi, and saprotrophic fungi per sample was significantly higher in soils than in roots (Fig. S5). Community composition of total fungi, EcM fungi, endophytic fungi, and saprotrophic fungi was significantly differentiated between roots and soils (Fig. S6).

### Diversity patterns along the elevation gradient

**Fig. 2:**
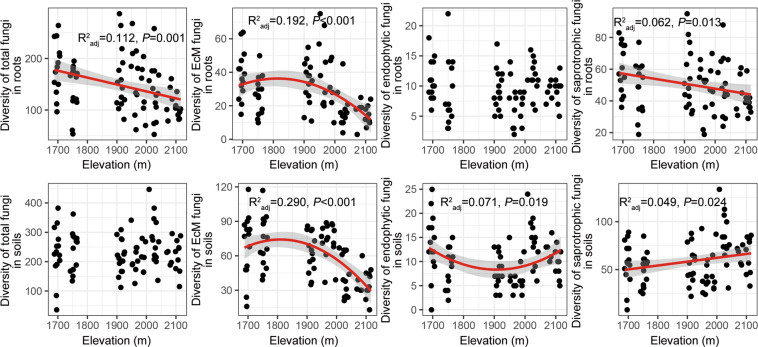
The elevation pattern in diversities of total fungi, EcM fungi, endophytic fungi, and saprotrophic fungi in roots and soils. The solid red lines indicate statistical significance for the relationships of diversities and elevation, and the shaded areas show the 95% confidence interval of the fit. *n* = 84 in either roots and soils.

Diversity of total fungi in roots declined monotonically with increasing elevation, whereas diversity of total fungi in soils did not vary significantly with elevation (Fig. 2). Diversity of EcM fungi decreased significantly with increasing elevation in roots and soils, with quadratic models fitting better than linear models (Fig. 2, Table S3). Beyond the treeline (ca. 1950 m a.s.l.), EcM fungal diversity dramatically decreased with increasing elevation in roots and soils (Fig. 2). Diversity of saprotrophic fungi in soils significantly increased with increasing elevation, whereas diversity of saprotrophic fungi in roots significantly decreased with increasing elevation (Fig. 2). Diversity of endophytic fungi in soils showed a U-shaped pattern with increasing elevation, whereas diversity of endophytic fungi in roots did not vary significantly with elevation (Fig. 2). LMMs showed the same elevation pattern for diversities of total fungi, EcM fungi, and saprotrophic fungi, as described above, when accounting for spatial effects (Table S4).

### Application of IBT on EcM fungal diversity

**Fig. 3:**
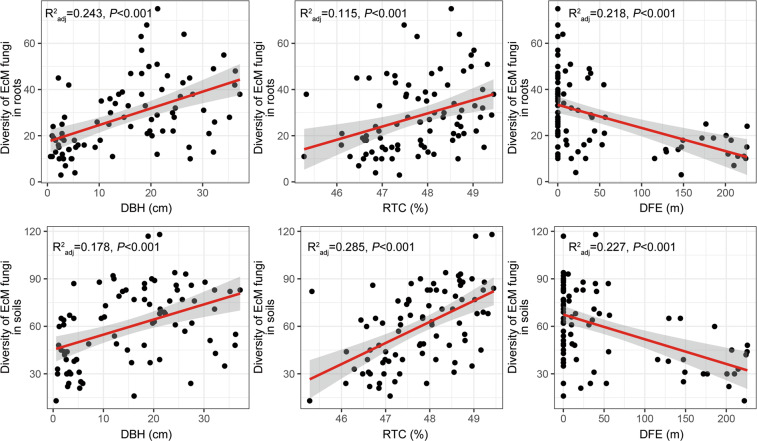
Island biogeography theory applies to EcM fungal diversity in roots and soils. The solid red lines indicate statistical significance for the relationships of EcM fungal diversity and island area (i.e., DBH), island energy (i.e., RTC) and island isolation (i.e., DFE), respectively, and the shaded areas show the 95% confidence interval of the fit. *n* = 84 in either roots and soils.

Out of 52 candidate variables (incl. soil properties, roots traits, DBH, tree height, canopy diameter, elevation, slope, and DFE), DBH and RTC were most strongly correlated with EcM fungal diversity in roots and soils, respectively (Table S5). EcM fungal diversity monotonically increased with increasing DBH and RTC but declined with increasing DFE in both roots and soils (Fig. 3). LMMs showed the same diversity-DBH, diversity-RTC, and diversity-DFE relationships for EcM fungi when accounting for spatial effects (Table S6). Here, DBH, RTC, and DFE represented the area, energy, and isolation of “virtual islands”, respectively, based on the IBT framework. In addition, the variation of EcM fungal diversity in relation to DBH more strongly conformed to power functions in roots and soils (Roots: *S* = 13.2709A^0.3085^, Soils: *S* = 37.2492A^0.1965^, Fig. S7).

According to the OLS multiple regression models #1, DBH (positive effect: Estimate = 0.28, *R*^2^_adj.partial_ = 0.243), FFA (positive effect: Estimate = 0.20, *R*^2^_adj.partial_ = 0.049), conductivity (negative effect: Estimate = −0.28, *R*^2^_adj.partial_ = 0.028) and available Mn (positive effect: Estimate = 0.19, *R*^2^_adj.partial_ = 0.029) were the best predictors of EcM fungal diversity in roots, cumulatively explaining 34.9% of the variation (AIC = −31.27). RTC (positive effect: Estimate = 0.25, *R*^2^_adj.partial_ = 0.285), cellulose (positive effect: Estimate = 0.30, *R*^2^_adj.partial_ = 0.086), PCNM5 (negative effect: Estimate = −0.22, *R*^2^_adj.partial_ = 0.037) and conductivity (negative effect: Estimate = −0.21, *R*^2^_adj.partial_ = 0.025) were the best predictors of EcM fungal diversity in soils, cumulatively explaining 43.3% of the variation (AIC = −42.79; Table 1).

**Table 1:** Summary of the best ordinary least squares (OLS) multiple regression models #1 for the effects of environmental variables and elevation gradient on EcM fungal diversity in roots and soils.

Predictors	Estimate	*t* value	*P* value	VIF	*R*^2^_adj.cum_
Roots: df = 79, ${R}^{2}_{\mathrm{adj}}$ = 0.349, SE_resid_ = 0.807, *P* < 0.001, AIC = −31.27					
DBH	0.28	2.40	0.019	1.75	0.243
FAA	0.20	2.00	0.049	1.25	0.292
Conductivity	−0.28	−2.41	0.018	1.75	0.320
Available Mn	0.19	2.16	0.034	1.07	0.349
Soils: df = 79, ${R}^{2}_{\mathrm{adj}}\,$= 0.433, SE_resid_ = 0.753, *P* < 0.001, AIC = −42.79					
RTC	0.25	2.40	0.019	1.57	0.285
Cellulose	0.30	3.04	0.003	1.44	0.371
PCNM5	−0.22	−2.57	0.012	1.03	0.408
Conductivity	−0.21	−2.11	0.038	1.48	0.433

Notably, PCNM1-5, as the proxies of geographic effects, are also added in the models.

*AIC* Akaike’s information criterion, *VIF* variance inflation factor.

*n* = 84 samples in either roots and soils.

### Biotic interactions among fungal guilds

**Fig. 4:**
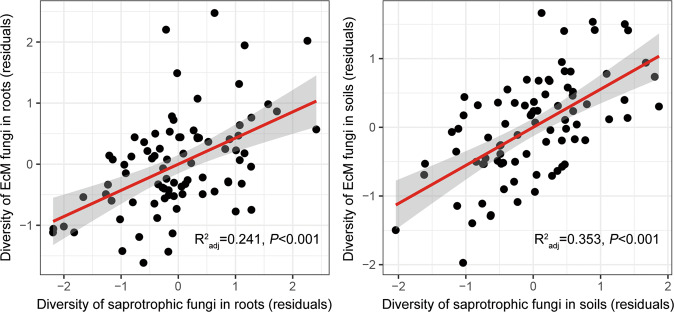
Relationships between EcM and saprotrophic fungal diversities in roots and soils when accounting for the effects of soil properties, roots traits, DBH, tree height, canopy diameter, elevation, slope, DFE, and spatial vectors. The residues of EcM and saprotrophic fungal diversities are fitted by PLSR. The solid red lines indicate statistical significance for the relationships, and the shaded areas show the 95% confidence interval of the fit. *n* = 84 in either roots and soils.

After adding diversities of endophytic and saprotrophic fungi as the candidate predictors, OLS multiple regression models #2 explained more variation in EcM fungal diversity with significant lower AIC values (Roots: *R*^2^_adj.cum_ = 0.514, AIC = −55.76; Soils: *R*^2^_adj.cum_ = 0.621, AIC = −74.74; Table 2). In roots, saprotrophic fungal diversity individually explained 31.3% of the variation in EcM fungal diversity (positive effect: Estimate = 0.45, *R*^2^_adj.partial_ = 0.313). Similarly, soil saprotrophic fungal diversity strongly affected the variation in EcM fungal diversity in soils (positive effect: Estimate = 0.48, *R*^2^_adj.partial_ = 0.088). The significant differences of OLS multiple regression models #1 and #2 were also corroborated by ANOVA tests (*P* < 0.001). Furthermore, PLSR analyses showed that increasing saprotrophic fungal diversity significantly enhanced EcM fungal diversity in roots and soils when accounting for all other significant predictive variables (Fig. 4). Similarly, greater number of EcM fungal lineages occurred with greater saprotrophic fungal richness at the genus, family, order, and class levels (Fig. S8).

**Table 2:** Summary of the best ordinary least squares (OLS) multiple regression models #2 for the effects of environmental variables, elevation gradient, and biotic interactions on EcM fungal diversity in roots and soils.

Predictors	Estimate	*t* value	*P* value	VIF	${{\mathbf{R}}^{2}_{adj.cum}}$
Roots: df = 79, ${R}^{2}_{\mathrm{adj}}$ = 0.514, SE_resid_ = 0.697, *P* < 0.001, AIC = −55.76					
Diversity of saprotrophic fungi in roots	0.45	5.66	<0.001	1.07	0.313
DBH	0.26	2.66	0.009	1.69	0.457
Conductivity	−0.27	−2.73	0.008	1.70	0.486
Available Mn	0.19	2.36	0.021	1.06	0.514
Soils: df = 77, ${R}^{2}_{\mathrm{adj}}$ = 0.621, SE_resid_ = 0.616, *P* < 0.001, AIC = −74.74					
RTC	0.23	2.55	0.013	1.71	0.285
Diversity of saprotrophic fungi in soils	0.48	6.14	<0.001	1.36	0.373
Conductivity	−0.49	−5.28	<0.001	1.92	0.532
Cellulose	0.25	3.05	0.003	1.46	0.571
DON	0.21	2.89	0.005	1.16	0.599
PCNM5	−0.16	−2.32	0.023	1.08	0.621

Notably, PCNM1-5, as the proxies of geographic effects, are also added in the models.

*AIC* Akaike’s information criterion, *VIF* variance inflation factor.

*n* = 84 samples in either roots and soils.

In addition, OLS multiple regression models showed that EcM and endophytic fungal diversities can positively affect saprotrophic fungal diversity in both roots and soils (Table S7). Saprotrophic fungal diversity can positively affect endophytic fungal diversity in roots and soils, respectively (Table S8).

### Integrated effects revealed by SEM

**Fig. 5:**
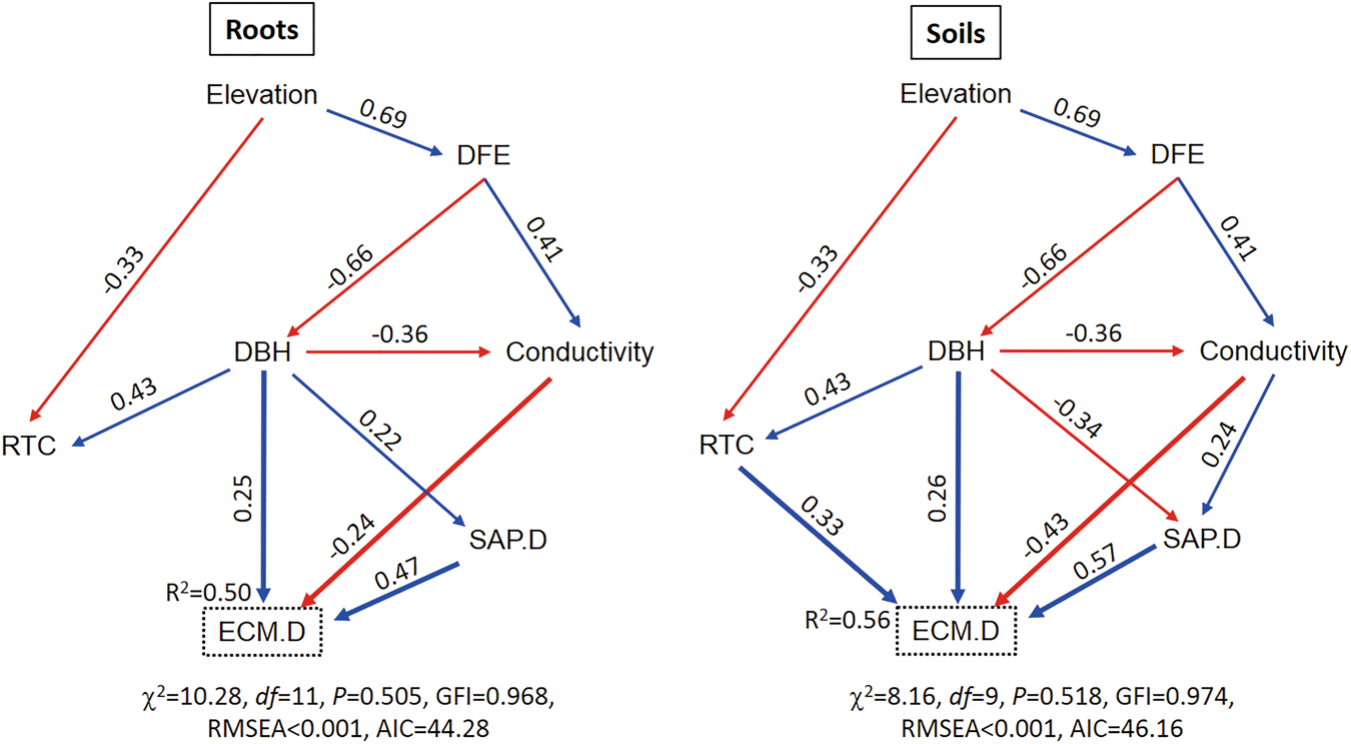
The best integrated SEM models revealing the direct and indirect effects of elevation, island isolation, island area, island energy, biotic interactions, and soil properties on EcM fungal diversity in roots and soils. DFE (distance to forest edge), DBH (diameter at breast height), RTC (root total carbon) and SAP.D (saprotrophic fungal diversity) in the diagrams served as proxies for island isolation, area, energy, and biotic interactions, respectively. The paths of direct effects on EcM fungal diversity are in bold, and only significant paths are retained. Blue color indicates the positive effect, while red color indicates the negative effect. SPC is shown near each corresponding path. GFI goodness of fit index, RMSEA root mean square error of approximation, ECM.D EcM fungal diversity. *n* = 84 in either roots and soils.

Based on the SEM theoretical model (Fig. S4), we built two and four SEM models for roots and soils, respectively. Although adding more predictors from OLS multiple regression models #2 into SEM enhanced predictive power of EcM fungal diversity, it notably increased AIC values by at least 12.10 and 9.36 in roots and soils, respectively (Figs. 5 and S9). Therefore, the best SEM models were the simplest models with lowest AIC values, which revealed the integrated effects of elevation, island area (i.e., DBH), island energy (i.e., RTC), island isolation (i.e., DFE), soil properties and biotic interactions (i.e., saprotrophic fungal diversity) on EcM fungal diversity. The best SEM models explained 50% and 56% of the variation of EcM fungal diversity in roots and soils, respectively (Fig. 5).

The positive or negative effect of each predictor in the best SEM models were the same as that in OLS multiple regression models and PLSR (Table 3). In both roots and soils, saprotrophic fungal diversity exhibited the strongest direct and total standardized effect on EcM fungal diversity (Roots: SPC = 0.470; Soils: SPC = 0.570). In addition, island isolation showed the strongest indirect effect on EcM fungal diversity in roots (SPC = −0.290), while elevation showed the strongest indirect effect on EcM fungal diversity in soils (SPC = −0.335). According to VPA, the independent effect on EcM fungal diversity was also strongest for saprotrophic fungal diversity either in roots or in soils (Fig. S10).

**Table 3:** Summary of the magnitude of each predictor on EcM fungal diversity by standardized path coefficient (SPC) in SEM models.

		Roots			Soils	
	Direct	Indirect	Total	Direct	Indirect	Total
Elevation	✗	−0.268	−0.268	✗	−0.335	−0.335
Island.I	✗	−0.290	−0.290	✗	−0.327	−0.327
Island.A	0.250	0.189	0.439	0.260	0.054	0.314
Island.E	✗	✗	✗	0.330	✗	0.330
Soil.P	−0.240	✗	−0.240	−0.430	0.137	−0.293
Biotic.I	0.470	✗	0.470	0.570	✗	0.570

Island.I, Island.A, and Island.E are represented by DFE, DBH, and RTC, respectively; Soil.P and Biotic.I are represented by soil conductivity and saprotrophic fungal diversity, respectively. These five variables and elevation consist of the best SEM models (Fig. 5), which explain the large variation of EcM fungal diversity. Here, ‘total’ indicates standardized total effect, which is summed by direct and indirect effects, and the symbol ‘✗’ indicates no significant effects.

## Discussion

### Elevation pattern of fungal diversity is niche- and guild-dependent

Along the 430-m vertical gradient, diversity of total fungi in roots decreased monotonically with increasing elevation, whereas diversity of total fungi in soils did not vary (Fig. 2). With increasing elevation, content of several nutrients in fine roots significantly decreased, for example cellulose (Pearson *r* = −0.69), hemicellulose (Pearson *r* = −0.78), and soluble sugar (Pearson *r* = −0.77). Smaller root systems predominated at the higher elevation sites. The reduced provision of nutrients and ‘shelters’ at higher elevations may lead to the monotonic decrease in total fungal diversity observed in roots but not in soils.

Saprotrophic fungal diversity monotonically increased with increasing elevation in soils (Fig. 2). Previously, at the similar elevation sites, we also observed that diversity of foliar fungal endophytes of *B. ermanii* significantly increased with increasing elevation.^16^ When leaves of *B. ermanii* fall, most endophytic fungi continue functioning as saprotrophs in litter and upper soil layers that may enhance the soil saprotrophic fungal diversity at higher elevations. Also, the higher diversity of herbaceous plants at the higher elevation habitats (data not shown) possibly contributed to the higher diversity of soil saprotrophic fungi by providing more diverse habitat and plant chemical inputs (i.e., the plant ‘Zinke’ effect).^78^ EcM fungal diversity significantly decreased with increasing elevation in both roots and soils (Fig. 2). This is consistent with studies in Hyrcanian forests of northern Iran and the Front Range of the Canadian Rockies,^8,79^ where climate factors were proposed as the primary mechanism to explain the variation. Like the responses of plant and animal diversities, harsher climate conditions such as lower temperature, stronger wind and associated seasonal water-deficiency may decrease EcM fungal diversity at higher elevations. Here, quadratic regression models better explained EcM fungal diversity than linear models (Tables S3, S4), and EcM fungal diversity decreased sharply beyond the treeline (Fig. 2), suggesting that alternative mechanisms may predict EcM fungal diversity besides the climate-driven hypothesis.

### Predicting EcM fungal diversity using the IBT framework

Consistent with our first hypothesis, EcM fungal diversity significantly increased with increasing DBH and RTC, but decreased with increasing DFE in both roots and soils (Fig. 3). These relationships remained when accounting for spatial effects (Table S6). DBH and RTC were also the strongest predictors of EcM fungal diversity in roots and soils, respectively, as revealed by Pearson correlation analyses (Table S5) and OLS multiple regression models #1 (Table 1). DBH is commonly used as a surrogate for tree size,^4,80^ and greater DBH reflects larger root systems of individual trees belowground.^81^

In terms of EcM fungal diversity in soils, RTC (rather than DBH) was the strongest predictor in the best OLS multiple regression model, exclusively explaining 28.5% of the variation (Table 1). The magnitude of island energy effect was much stronger than that of island area effect on EcM fungal diversity in neighboring soils. Wright (1983) proposed that species-energy theory, as the extension of IBT, could explain ~70–80% of the variation in diversities on islands.^22^ Interestingly, we also observed the correspondence of species-energy theory to foliar fungal endophytic diversity of *B. ermanii* in the same study area. In particular, diversity of foliar fungal endophytes strongly increased with increasing leaf total carbon content.^16^ These results collectively highlight the tight linkage between vegetation carbon pools and symbiotic fungal diversity within this timberline ecosystem, both aboveground and belowground.

In the present study, we showed the prevalence of IBT in predicting EcM fungal diversity along the timberline. Over the last several decades, some studies have used IBT to predict diversities of plant-associated fungi, e.g., phyllosphere fungi,^82^ nectar-inhabiting fungi,^83^ and AM fungi.^84^ Coincidentally, diversity of EcM fungi fitted well into the framework of IBT, exhibiting strong species-area and species-isolation relationships.^25–27^ This is partly attributed to the relatively strong dispersal limitation and high reliance of EcM fungi on plant carbon sources compared with other fungal guilds.^85^ In addition, we found that the relationship of EcM fungal diversity with DBH more strongly conformed to power functions, with the *z*-values (i.e., species-area slope) 0.31 and 0.20 in roots and soils, respectively (Fig. S7). These values are within the range reported for various macroorganism groups^21^ and agree with the values reported for EcM fungi previously.^25^ Furthermore, these values imply that EcM fungal diversity varies more in roots than that in soils with increasing island area. EcM fungal diversity in soils seems to be more affected by the island energy effect, in particular RTC and cellulose content (Tables 1 and S5).

### The positive effect of saprotrophic fungal diversity on EcM fungal diversity

Consistent with our second hypothesis, biotic interactions strongly contributed to predicting EcM fungal diversity in roots and soils. The OLS multiple regression models #2 and PLSR showed that saprotrophic fungal diversity significantly enhanced EcM fungal diversity in roots and soils when accounting for the effects of soil properties, roots traits, DBH, tree height, canopy diameter, elevation, slope, DFE, and spatial factors (i.e., PCNM1-5) (Table 2, Fig. 4).

Recently, intense competition between EcM fungi and saprotrophic fungi for nitrogen in organic materials (i.e., Gadgil effect) has been addressed.^86,87^ As such, limited shared resources and competitive exclusion are supposed to restrict the number of fungal taxa coexisting in the same niche. In this study, we observed a strong positive association between EcM and saprotrophic fungal diversities—increasing saprotrophic fungal diversity exclusively explained 31.3% and 8.8% of the increase in EcM fungal diversity in roots and soils, respectively (Table 2). We assume that it may be primarily attributed to the evolutionary source effect, as EcM fungi independently evolved ~60 times from different free-living saprotrophic fungal lineages.^2^ Here, the increasing numbers of saprotrophic fungal genera, families, orders, and classes also significantly enhanced the numbers of EcM fungal lineages in roots and soils (Fig. S8). It implies that the more lineages of saprotrophic fungi occur, the more lineages of EcM fungi are accompanied through the long evolutionary history, and this evolutionary association may reinforce co-dependencies in biodiversity.^88^

In the best OLS multiple regression models with saprotrophic fungal diversity as a response variable, increasing EcM fungal diversity similarly explained 31.3% and 4.9% of the increases in saprotrophic fungal diversity in roots and soils, respectively (Table S7). The reverse SEM models (Fig. S11) assuming that EcM fungal diversity directly affects saprotrophic fungal diversity also fitted equally well as the best SEM models (Fig. 5), with the difference of AIC values less than 2. That means that we cannot determine which direction of this biotic interaction is better, and the diversities of EcM and saprotrophic fungi may affect each other. Anyway, the determination on biotic interaction directions was out of the scope of this study based on the present data.

### Integrative modeling: comprehensive understanding with multilevel frameworks

As the integrative modeling in this study, SEM revealed the significant effects of elevation, elements within IBT (i.e., island area, energy, and isolation), biotic interactions and soil properties on EcM fungal diversities in roots and soils (Fig. 5). Furthermore, SEM discerned the direct and indirect effects of multilevel predictors and their relative contributions, respectively (Table 3). In particular, the integrative modeling reveals some cryptic mechanisms that are hardly found by simple models (e.g., traditional bivariate analyses). One study on relationships between plant richness and productivity detailed the advantages of integrative modeling and pointed out a strong and consistent enhancement of productivity by richness that was in striking contrast with the superficial data patterns.^39^ In our study, the effect of saprotrophic fungal diversity on EcM fungal diversity was not seen in soils by bivariate regressions (*R*^2^_adj_ = 0.012, *P* = 0.163). However, SEM clearly showed that this effect of saprotrophic fungal diversity was direct and strongest in soils (Table 3). In addition, SEM revealed the indirect effects of elevation and DFE in roots and soils, although these two effects were absent in the best OLS multiple regression models (Table 2).

One of the major findings revealed by SEM may be the extremely strong and direct effect of saprotrophic fungal diversity on EcM fungal diversity in both roots and soils. This implies the pronounced role of biotic interactions in predicting EcM fungal diversity pattern in this relict and timberline environment, and diversities of saprotrophic and EcM fungi rely on each other that may be through a complex ecological and evolutionary linkage. Nonetheless, there were still fine-scale distinctions in the relative contributions of different predictors between roots and soils. For example, island isolation showed the strongest indirect effect on EcM fungal diversity in roots, while elevation showed the strongest indirect effect on EcM fungal diversity in soils; island area was the second strongest factor in terms of direct effect in roots, while soil properties was the second strongest factor in soils (Table 3). Similarly, VPA showed that elevation and soil properties accumulatively explained 39.7% of the variation in EcM fungal diversity in soils, while they only explained 24.3% of the variation in roots (Fig. S10). Climate and soil factors seem to be more important in affecting EcM fungal diversity in soil environment than fine roots.

## Conclusion

By means of integrative modeling and multiple models comparisons, we revealed the consistent and pronounced effects of both the elements within IBT and saprotrophic fungal diversity on EcM fungal diversity of *B. ermanii* in both roots and soils. Recently, the concept has emerged that regional EcM fungal diversity pattern will be deeply impacted by warming of forests in future,^89^ and low diversity and availability of EcM fungal taxa beyond the range edge of host plant may conversely limit the range expansion of that plant under climate change scenario.^52^ Considering the warming-induced upwards migration of treeline on Changbai Mountain,^51^ EcM fungal diversity of *B. ermanii* will suffer from a dramatic shift across this subalpine landscape in future. Our findings suggest that their shift will strongly depend on the diversities of other fungal guilds (e.g., saprotrophic fungi in soils) and vary between different niches (fine roots vs. neighboring soils), together giving a feedback to climate change and landscape vegetation dynamics through fungal-associated biogeochemical processes.^90,91^

## Supplementary information

Supplementary Information

## Data Availability

The sequence data associated with this study were submitted to the European Nucleotide Archive under the accession number PRJEB34192. Environmental and geographic data are deposited in the Dryad digital repository (10.5061/dryad.2280gb5rk).

## References

[1] Tedersoo L (2014). Fungal biogeography. Global diversity and geography of soil fungi. Science..

[2] Martin F, Kohler A, Murat C, Veneault-Fourrey C, Hibbett DS (2016). Unearthing the roots of ectomycorrhizal symbioses. Nat Rev Microbiol..

[3] Lindahl BD, Tunlid A (2015). Ectomycorrhizal fungi - potential organic matter decomposers, yet not saprotrophs. New Phytol..

[4] Chen L (2019). Differential soil fungus accumulation and density dependence of trees in a subtropical forest. Science..

[5] Tedersoo L, Bahram M, Zobel M (2020). How mycorrhizal associations drive plant population and community biology. Science..

[6] Jansson JK, Hofmockel KS (2020). Soil microbiomes and climate change. Nat Rev Microbiol..

[7] Stevens GC (1992). The elevational gradient in altitudinal range: an extension of Rapoport’s latitudinal rule to altitude. Am Nat..

[8] Bahram M, Polme S, Koljalg U, Zarre S, Tedersoo L (2012). Regional and local patterns of ectomycorrhizal fungal diversity and community structure along an altitudinal gradient in the Hyrcanian forests of northern Iran. New Phytol..

[9] Miyamoto Y, Nakano T, Hattori M, Nara K (2014). The mid-domain effect in ectomycorrhizal fungi: range overlap along an elevation gradient on Mount Fuji, Japan. ISME J..

[10] Han QS, Huang J, Long DF, Wang XB, Liu JJ (2017). Diversity and community structure of ectomycorrhizal fungi associated with Larix chinensis across the alpine treeline ecotone of Taibai Mountain. Mycorrhiza..

[11] Jarvis SG, Woodward S, Taylor AFS (2015). Strong altitudinal partitioning in the distributions of ectomycorrhizal fungi along a short (300 m) elevation gradient. New Phytol..

[12] Nottingham AT (2018). Microbes follow Humboldt: temperature drives plant and soil microbial diversity patterns from the Amazon to the Andes. Ecology..

[13] Harrison S, Spasojevic MJ, Li DJ (2020). Climate and plant community diversity in space and time. Proc Natl Acad Sci USA..

[14] Arnold AE, Lutzoni F (2007). Diversity and host range of foliar fungal endophytes: Are tropical leaves biodiversity hotspots?. Ecology..

[15] Newsham KK (2016). Relationship between soil fungal diversity and temperature in the maritime Antarctic. Nat Clim Change..

[16] Yang T (2016). Carbon constrains fungal endophyte assemblages along the timberline. Environ. Microbiol..

[17] Ishida TA, Nara K, Hogetsu T (2007). Host effects on ectomycorrhizal fungal communities: insight from eight host species in mixed conifer-broadleaf forests. New Phytol..

[18] Suz LM (2014). Environmental drivers of ectomycorrhizal communities in Europe’s temperate oak forests. Mol Ecol..

[19] Truong C (2019). Ectomycorrhizal fungi and soil enzymes exhibit contrasting patterns along elevation gradients in southern Patagonia. New Phytol..

[20] Koizumi T, Nara K (2020). Ectomycorrhizal fungal communities in ice-age relict forests of Pinus pumila on nine mountains correspond to summer temperature. ISME J..

[21] MacArthur RH, Wilson EO (1967). The Theory of Island Biogeography..

[22] Wright DH (1983). Species-energy theory: an expansion of species-area theory. Oikos..

[23] Li SP (2020). Island biogeography of soil bacteria and fungi: similar patterns, but different mechanisms. ISME J..

[24] Janzen DH (1968). Host Plants as Islands in Evolutionary and Contemporary Time. Am Nat..

[25] Peay KG, Bruns TD, Kennedy PG, Bergemann SE, Garbelotto M (2007). A strong species-area relationship for eukaryotic soil microbes: island size matters for ectomycorrhizal fungi. Ecol Lett..

[26] Peay KG, Garbelotto M, Bruns TD (2010). Evidence of dispersal limitation in soil microorganisms: Isolation reduces species richness on mycorrhizal tree islands. Ecology..

[27] Glassman SI, Lubetkin KC, Chung JA, Bruns TD (2017). The theory of island biogeography applies to ectomycorrhizal fungi in subalpine tree “islands” at a fine scale. Ecosphere..

[28] Yang T (2019). Phylogenetic imprint of woody plants on the soil mycobiome in natural mountain forests of eastern China. ISME J..

[29] Toju H, Guimaraes PR, Olesen JM, Thompson JN (2014). Assembly of complex plant-fungus networks. Nat Commun..

[30] Toju H, Guimaraes PR, Olesen JM, Thompson JN (2015). Below-ground plant-fungus network topology is not congruent with above-ground plant-animal network topology. Sci Adv..

[31] Tedersoo, L., Smith, M. E. Ectomycorrhizal fungal lineages: detection of four new groups and notes on consistent recognition of ectomycorrhizal taxa in high-throughput sequencing studies. In: *Biogeography of mycorrhizal symbiosis* (ed. Tedersoo, L.). 125–142. (Springer International Publishing, 2017).

[32] Miyauchi S (2020). Large-scale genome sequencing of mycorrhizal fungi provides insights into the early evolution of symbiotic traits. Nat Commun..

[33] Smith GR, Finlay RD, Stenlid J, Vasaitis R, Menkis A (2017). Growing evidence for facultative biotrophy in saprotrophic fungi: data from microcosm tests with 201 species of wood-decay basidiomycetes. New Phytol..

[34] Schulz B, Boyle C (2005). The endophytic continuum. Mycol Res..

[35] Rodriguez RJ, White JF, Arnold AE, Redman RS (2009). Fungal endophytes: diversity and functional roles. New Phytol..

[36] Strullu-Derrien C, Selosse MA, Kenrick P, Martin FM (2018). The origin and evolution of mycorrhizal symbioses: from palaeomycology to phylogenomics. New Phytol..

[37] Botnen S (2014). Low host specificity of root-associated fungi at an Arctic site. Mol Ecol..

[38] Almario J (2017). Root-associated fungal microbiota of nonmycorrhizal *Arabis alpina* and its contribution to plant phosphorus nutrition. Proc Natl Acad Sci USA..

[39] Grace JB (2016). Integrative modelling reveals mechanisms linking productivity and plant species richness. Nature..

[40] Takahashi K, Tokumitsu Y, Yasue K (2005). Climatic factors affecting the tree-ring width of *Betula ermanii* at the timberline on Mount Norikura, central Japan. Ecol Res..

[41] Sano M, Furuta F, Sweda T (2010). Summer temperature variations in southern Kamchatka as reconstructed from a 247-year tree-ring chronology of *Betula ermanii*. J Forest Res..

[42] Wang XM, Zhao XH, Gao LS (2013). Climatic response of *Betula ermanii* along an altitudinal gradient in the northern slope of Changbai Mountain. China. Dendrobiology..

[43] Gansert D, Backes K, Kakubari Y (1999). Altitudinal and seasonal variation of frost resistance of *Fagus crenata* and *Betula ermanii* along the Pacific slope of Mt. Fuji, Japan. J Ecol..

[44] Toda M (2018). Photosynthetically distinct responses of an early-successional tree, *Betula ermanii*, following a defoliating disturbance: observational results of a manipulated typhoon-mimic experiment. Trees-Struct Funct..

[45] Cong Y (2019). Decreased temperature with increasing elevation decreases the end-season leaf-to-wood reallocation of resources in deciduous *Betula ermanii* Cham. trees. Forests..

[46] Osono T, Hirose D (2009). Altitudinal distribution of microfungi associated with *Betula ermanii* leaf litter on Mt. Rishiri, northern Japan. Can J Microbiol..

[47] Nara K, Hogetsu T (2004). Ectomycorrhizal fungi on established shrubs facilitate subsequent seedling establishment of successional plant species. Ecology..

[48] Yu DP, Wang GG, Dai LM, Wang QL (2007). Dendroclimatic analysis of *Betula ermanii* forests at their upper limit of distribution in Changbai Mountain, Northeast China. Forest Ecol Manag..

[49] Bai F (2008). Long-term protection effects of national reserve to forest vegetation in 4 decades: biodiversity change analysis of major forest types in Changbai Mountain Nature Reserve, China. Sci China Ser C..

[50] Bai F, Sang WG, Axmacher JC (2011). Forest vegetation responses to climate and environmental change: a case study from Changbai Mountain, NE China. Forest Ecol Manag..

[51] Du HB (2018). Warming-induced upward migration of the alpine treeline in the Changbai Mountains, northeast China. Global Change Biol..

[52] Lankau RA, Keymer DP (2016). Ectomycorrhizal fungal richness declines towards the host species’ range edge. Mol Ecol..

[53] Zbiral J, Nemec P (2000). Integrating of Mehlich 3 extractant into the Czech soil testing scheme. Commun Soil Sci Plan..

[54] Bradford MM, Williams WL (1976). New, Rapid, Sensitive method for protein determination. Fed Proc..

[55] Hansen MC (2013). High-resolution global maps of 21st-century forest cover change. Science..

[56] Lund HG (2002). When is a forest not a forest?. J Forest..

[57] Magoc T, Salzberg SL (2011). FLASH: fast length adjustment of short reads to improve genome assemblies. Bioinformatics..

[58] Caporaso JG (2010). QIIME allows analysis of high-throughput community sequencing data. Nat Methods..

[59] Martin M (2011). Cutadapt removes adapter sequences from high-throughput sequencing reads. EMBnet J..

[60] Bengtsson-Palme J (2013). Improved software detection and extraction of ITS1 and ITS2 from ribosomal ITS sequences of fungi and other eukaryotes for analysis of environmental sequencing data. Methods Ecol Evol..

[61] Edgar RC, Haas BJ, Clemente JC, Quince C, Knight R (2011). UCHIME improves sensitivity and speed of chimera detection. Bioinformatics..

[62] Edgar RC (2010). Search and clustering orders of magnitude faster than BLAST. Bioinformatics.

[63] Wang Q, Garrity GM, Tiedje JM, Cole JR (2007). Naive Bayesian classifier for rapid assignment of rRNA sequences into the new bacterial taxonomy. Appl Environ Microb..

[64] Koljalg U (2013). Towards a unified paradigm for sequence-based identification of fungi. Mol. Ecol..

[65] Froslev TG (2017). Algorithm for post-clustering curation of DNA amplicon data yields reliable biodiversity estimates. Nat. Commun..

[66] Nguyen NH (2016). FUNGuild: An open annotation tool for parsing fungal community datasets by ecological guild. Fungal Ecol..

[67] R Core Team. R: A language and environment for statistical computing. R Foundation for Statistical Computing, Vienna, Austria. URL https://www.R-project.org/. 2018.

[68] Yang T (2017). Soil fungal diversity in natural grasslands of the Tibetan Plateau: associations with plant diversity and productivity. New Phytol..

[69] Bates D, Mächler M, Bolker B, Walker S (2015). Fitting Linear Mixed-Effects Models Using lme4. J Stat Softw..

[70] Fox J, Weisberg S (2019). An R Companion to Applied Regression.

[71] Bartoń K. MuMIn: Multi-Model Inference. R package version 1.43.6. https://CRAN.R-project.org/package=MuMIn. 2019

[72] Gleason HA (1925). Species and Area. Ecology..

[73] Borcard D, Legendre P (2002). All-scale spatial analysis of ecological data by means of principal coordinates of neighbour matrices. Ecol Model..

[74] Venables WN, Ripley BD (2002). Modern Applied Statistics with S..

[75] Dray S., Legendre P., Blanchet F. G. Packfor: forward selection with permutation (Canoco p. 46). R package version 0.0-8/r136. https://RForge.R-project.org/projects/sedar/. 2016.

[76] Mevik B. H., Wehrens R., Liland K. H. pls: Partial Least Squares and Principal Component Regression. R package version 2.7-2. https://CRAN.R-project.org/package=pls. 2019.

[77] Oksanen J., et al. VEGAN: Community ecology package. R package v.2.5-3. [WWW document] URL http://CRAN.R-project.org/package=vegan. 2018.

[78] Waring BG (2015). Pervasive and strong effects of plants on soil chemistry: a meta-analysis of individual plant ‘Zinke’ effects. P Roy Soc B-Biol Sci..

[79] Kernaghan G, Harper KA (2001). Community structure of ectomycorrhizal fungi across an alpine/subalpine ecotone. Ecography..

[80] Wang CK (2006). Biomass allometric equations for 10 co-occurring tree species in Chinese temperate forests. Forest Ecol Manag..

[81] Kachamba DJ, Eid T, Gobakken T (2016). Above- and Belowground Biomass Models for Trees in the Miombo Woodlands of Malawi. Forests..

[82] Andrews JH, Kinkel LL, Berbee FM, Nordheim EV (1987). Fungi, Leaves, and the Theory of Island Biogeography. Microbial Ecol..

[83] Belisle M, Peay KG, Fukami T (2012). Flowers as Islands: Spatial Distribution of Nectar-Inhabiting Microfungi among Plants of *Mimulus aurantiacus*, a Hummingbird-Pollinated Shrub. Microbial Ecol..

[84] Camargo-Ricalde SL, Dhillion SS (2003). Endemic Mimosa species can serve as mycorrhizal “resource islands” within semiarid communities of the Tehuacan-Cuicatlan Valley, Mexico. Mycorrhiza..

[85] Tedersoo L, Bahram M (2019). Mycorrhizal types differ in ecophysiology and alter plant nutrition and soil processes. Biol Rev..

[86] Smith GR, Wan J (2019). Resource-ratio theory predicts mycorrhizal control of litter decomposition. New Phytol..

[87] Zak DR (2019). Exploring the role of ectomycorrhizal fungi in soil carbon dynamics. New Phytol..

[88] Castledine M, Sierocinski P, Padfield D, Buckling A (2020). Community coalescence: an eco-evolutionary perspective. Philos Trans R Soc Lond B Biol Sci..

[89] Steidinger BS (2020). Ectomycorrhizal fungal diversity predicted to substantially decline due to climate changes in North American Pinaceae forests. J Biogeogr..

[90] Crowther TW (2019). The global soil community and its influence on biogeochemistry. Science..

[91] Zanne AE (2020). Fungal functional ecology: bringing a trait-based approach to plant-associated fungi. Biol Rev..

