# Supplementary material for: Saprotrophic fungal diversity predicts ectomycorrhizal fungal diversity along the timberline in the framework of island biogeography theory
Source: ISME Commun. 2021 May 18;1:15. doi: 10.1038/s43705-021-00015-1 (PMC9723781; doi:10.1038/s43705-021-00015-1)
Supplement: Supplementary file 1 — Supplementary Information [file 43705_2021_15_MOESM1_ESM.docx]

**Supplementary Information**

**Title:** Saprotrophic fungal diversity predicts ectomycorrhizal fungal diversity along the timberline in the framework of island biogeography theory

**Running title:** Associations of saprotrophic and ectomycorrhizal fungal diversities

Teng Yang^1,2^, Leho Tedersoo^3,4^, Xiao Fu^1,2^, Chang Zhao^5^, Xu Liu^1,2^, Guifeng Gao^1^, Liang Cheng^1,2^, Jonathan M. Adams^6^, Haiyan Chu^1,2*^

^1^State Key Laboratory of Soil and Sustainable Agriculture, Institute of Soil Science, Chinese Academy of Sciences, East Beijing Road 71, Nanjing 210008, China

^2^University of Chinese Academy of Sciences, Beijing 100049, China

^3^Mycology and Microbiology Center, University of Tartu, 14a Ravila, Tartu 50411, Estonia

^4^College of Science, King Saud University, Riyadh, 11451, Saudi Arabia

^5^School of Geography Sciences, Nanjing Normal University, Nanjing 210023, China

^6^School of Geographic and Oceanographic Sciences, Nanjing University, Nanjing 210023, China

**^*^Corresponding author**: Haiyan Chu

Tel: +86 02586881356, E-mail: [hychu@issas.ac.cn](mailto:hychu@issas.ac.cn)

Table S1 – Table S8

Fig. S1 – Fig. S11

**Table S1** Assignments of 6663 fungal OTUs to functional guilds in this study.

| Functional guilds | OTU No. | Sequence No. | Proportion (%) | Frequency (Sample No.) |
| --- | --- | --- | --- | --- |
| EcM fungi | 1056 | 3,296,818 | 42.0 | 168 |
| Endophytic fungi | 277 | 439,340 | 5.6 | 168 |
| ErM fungi | 36 | 21,994 | 0.3 | 155 |
| Lichenized fungi | 94 | 10,279 | 0.1 | 156 |
| Plant pathogens | 254 | 38,667 | 0.5 | 166 |
| Saprotrophic fungi | 1871 | 2,172,118 | 27.7 | 168 |
| Unknown | 3075 | 1,869,910 | 23.8 | 168 |
| Total | 6663 | 7,849,126 | 100.0 | 168 |

Due to the small proportion of sequences (< 0.1%), animal pathogens, arbuscular mycorrhizal fungi, fungal parasites and lichen parasites are classified to unknown for clarity. n=84 samples in either roots and soils.

**Table S2** The relative abundances of EcM lineages in different elevation sites and niches.

| Relative abundance (%) | Roots | | | | | | | Soils | | | | | | |
| --- | --- | --- | --- | --- | --- | --- | --- | --- | --- | --- | --- | --- | --- | --- |
|  | UL | TI | TL | PS | DCEB | DL | All | UL | TI | TL | PS | DCEB | DL | All |
| /acephala macrosclerotiorum | 0.5 | 0.6 | 0.0 | 0.0 | 0.2 | 0.0 | 0.1 | 1.4 | 0.4 | 0.0 | 0.0 | 0.0 | 0.0 | 0.1 |
| /aleurina | 0.0 | 0.0 | 0.0 | 0.0 | 0.0 | 0.0 | 0.0 | 0.0 | 0.0 | 0.0 | 0.0 | 0.0 | 0.0 | 0.0 |
| /amanita | 0.0 | 0.0 | 0.1 | 1.1 | 0.1 | 1.3 | 0.5 | 0.0 | 0.0 | 1.3 | 2.0 | 0.2 | 0.6 | 1.0 |
| /amphinema-tylospora | 0.4 | 0.1 | 0.2 | 0.0 | 0.0 | 1.5 | 0.4 | 2.9 | 0.7 | 1.2 | 0.4 | 1.9 | 2.8 | 1.6 |
| /boletus | 14.4 | 0.3 | 0.0 | 0.0 | 0.0 | 0.2 | 1.3 | 1.1 | 0.4 | 0.1 | 0.0 | 0.0 | 0.2 | 0.1 |
| /byssocorticium | 0.0 | 0.0 | 0.0 | 0.2 | 0.0 | 0.0 | 0.0 | 0.0 | 0.0 | 0.8 | 0.2 | 0.0 | 0.9 | 0.5 |
| /cantharellus | 0.0 | 0.0 | 0.0 | 0.0 | 0.0 | 0.1 | 0.0 | 0.5 | 1.8 | 0.2 | 0.1 | 0.8 | 10.8 | 3.0 |
| /cenococcum | 0.0 | 0.0 | 0.0 | 0.0 | 0.0 | 0.0 | 0.0 | 0.0 | 0.0 | 0.0 | 0.0 | 0.0 | 0.0 | 0.0 |
| /clavulina | 0.0 | 7.2 | 0.9 | 3.7 | 7.0 | 6.4 | 3.8 | 3.2 | 3.9 | 0.8 | 4.0 | 3.8 | 3.5 | 3.0 |
| /cortinarius | 6.9 | 9.9 | 9.1 | 6.7 | 6.4 | 5.0 | 7.3 | 6.5 | 3.5 | 17.5 | 8.7 | 9.1 | 2.3 | 9.0 |
| /elaphomyces | 0.0 | 0.0 | 0.1 | 1.7 | 0.0 | 0.0 | 0.4 | 0.0 | 0.0 | 0.9 | 0.9 | 1.4 | 1.5 | 1.1 |
| /entoloma | 0.0 | 3.8 | 0.1 | 1.5 | 1.2 | 0.0 | 0.8 | 0.2 | 5.0 | 3.0 | 12.6 | 0.8 | 0.6 | 4.4 |
| /galactinia | 0.0 | 0.7 | 0.0 | 0.0 | 0.0 | 0.1 | 0.1 | 0.0 | 1.4 | 0.1 | 0.0 | 0.0 | 0.1 | 0.1 |
| /genea-humaria | 0.0 | 0.0 | 0.0 | 0.2 | 0.3 | 0.4 | 0.2 | 0.0 | 0.0 | 0.0 | 0.1 | 0.7 | 0.3 | 0.2 |
| /geopora | 0.0 | 0.0 | 0.0 | 0.0 | 0.0 | 0.0 | 0.0 | 0.0 | 0.0 | 0.0 | 0.0 | 0.0 | 0.0 | 0.0 |
| /hebeloma-alnicola | 0.4 | 3.9 | 0.1 | 0.0 | 0.5 | 0.2 | 0.4 | 0.2 | 1.6 | 0.1 | 0.4 | 0.9 | 1.8 | 0.8 |
| /hyaloscypha | 0.0 | 0.2 | 0.1 | 0.1 | 0.1 | 0.3 | 0.1 | 1.6 | 0.5 | 0.1 | 0.1 | 0.1 | 0.1 | 0.2 |
| /hydnotrya | 0.0 | 0.0 | 0.0 | 0.0 | 0.0 | 0.0 | 0.0 | 0.0 | 0.0 | 0.0 | 0.0 | 0.0 | 0.0 | 0.0 |
| /hygrophorus | 0.0 | 0.0 | 0.0 | 0.0 | 0.0 | 0.9 | 0.1 | 0.0 | 0.0 | 0.0 | 1.5 | 15.0 | 0.0 | 3.1 |
| /inocybe | 0.0 | 0.5 | 0.7 | 0.0 | 1.3 | 0.3 | 0.5 | 12.3 | 6.9 | 7.9 | 4.4 | 3.8 | 6.6 | 6.0 |
| /laccaria | 0.3 | 0.8 | 0.3 | 2.4 | 0.2 | 4.4 | 1.5 | 4.6 | 1.8 | 0.4 | 2.5 | 0.7 | 4.6 | 2.2 |
| /otidea | 0.0 | 0.0 | 0.0 | 0.0 | 0.0 | 0.1 | 0.0 | 0.0 | 0.0 | 0.0 | 0.0 | 0.0 | 0.1 | 0.0 |
| /paralyophyllum | 0.0 | 0.0 | 0.0 | 0.0 | 0.0 | 0.0 | 0.0 | 0.0 | 0.0 | 0.0 | 0.0 | 0.0 | 0.0 | 0.0 |
| /paxillus-gyrodon | 0.0 | 0.0 | 0.0 | 0.0 | 0.0 | 0.0 | 0.0 | 0.0 | 0.0 | 0.0 | 0.0 | 0.0 | 0.0 | 0.0 |
| /piloderma | 3.8 | 0.0 | 11.5 | 8.9 | 2.9 | 32.3 | 11.7 | 6.1 | 0.0 | 0.4 | 5.5 | 1.5 | 13.8 | 5.3 |
| /pseudotomentella | 0.4 | 0.0 | 0.3 | 3.2 | 0.1 | 2.6 | 1.3 | 0.1 | 0.1 | 0.2 | 0.5 | 0.1 | 1.9 | 0.6 |
| /rhodoscypha | 0.0 | 0.0 | 0.0 | 0.0 | 0.0 | 0.0 | 0.0 | 0.0 | 0.0 | 0.0 | 0.0 | 0.0 | 0.0 | 0.0 |
| /russula-lactarius | 39.3 | 10.6 | 70.0 | 66.4 | 60.6 | 34.9 | 55.0 | 32.4 | 27.6 | 56.6 | 41.5 | 37.9 | 40.2 | 43.3 |
| /sebacina | 0.0 | 0.5 | 0.0 | 0.0 | 0.6 | 1.9 | 0.5 | 0.7 | 9.3 | 3.6 | 2.1 | 3.1 | 3.3 | 3.3 |
| /serendipita1 | 0.9 | 1.2 | 0.1 | 0.0 | 0.0 | 0.0 | 0.2 | 10.5 | 8.4 | 0.2 | 0.3 | 0.1 | 0.1 | 0.9 |
| /sphaerosporella | 0.0 | 0.0 | 0.0 | 0.0 | 0.0 | 0.0 | 0.0 | 0.0 | 0.0 | 0.0 | 0.0 | 0.0 | 0.0 | 0.0 |
| /suillus-rhizopogon | 0.0 | 0.0 | 0.0 | 0.0 | 0.0 | 0.1 | 0.0 | 0.0 | 0.0 | 0.0 | 0.0 | 0.0 | 0.0 | 0.0 |
| /tarzetta | 0.0 | 0.0 | 0.0 | 0.0 | 0.0 | 0.0 | 0.0 | 0.0 | 0.0 | 0.1 | 0.1 | 0.0 | 0.0 | 0.0 |
| /tomentella-thelephora | 27.5 | 54.8 | 6.3 | 3.7 | 18.3 | 6.7 | 12.9 | 15.5 | 26.3 | 4.6 | 12.0 | 17.0 | 3.0 | 9.7 |
| /tomentellopsis | 4.6 | 0.4 | 0.0 | 0.1 | 0.1 | 0.0 | 0.5 | 0.0 | 0.1 | 0.0 | 0.0 | 0.7 | 0.3 | 0.2 |
| /tricholoma | 0.0 | 0.0 | 0.0 | 0.0 | 0.0 | 0.0 | 0.0 | 0.0 | 0.0 | 0.0 | 0.0 | 0.0 | 0.1 | 0.0 |
| /tuber-helvella | 0.5 | 4.4 | 0.0 | 0.0 | 0.0 | 0.2 | 0.4 | 0.0 | 0.1 | 0.0 | 0.0 | 0.0 | 0.0 | 0.0 |
| /wilcoxina | 0.0 | 0.0 | 0.0 | 0.0 | 0.0 | 0.3 | 0.1 | 0.0 | 0.0 | 0.0 | 0.0 | 0.2 | 0.5 | 0.2 |

UL: upper limit (2069-2116 m); TI: tree island (1997-2042 m); TL: treeline (1949-1992 m); PS: pure stands (1900-1926 m); DCEB: ecotone of dark coniferous forests and Erman’s birch forests (1742-1765 m); DL: down limit (1688-1706). All indicates the sums of all elevations in roots and soils, respectively.

**Table S3** The fitted regression models of fungal diversities along the elevation gradient.

|  | AIC.linear | *P*.linear | AIC.quadratic | *P*.quadratic |
| --- | --- | --- | --- | --- |
| **Roots:** |  |  |  |  |
| Diversity of total fungi | **665.38** | 0.001 | 665.41 | 0.002 |
| Diversity of EcM fungi | 453.33 | <0.001 | **448.11** | <0.001 |
| Diversity of endophytic fungi | **224.96** | 0.552 | 225.25 | 0.368 |
| Diversity of saprotrophic fungi | **470.50** | 0.013 | 472.25 | 0.041 |
| **Soils:** |  |  |  |  |
| Diversity of total fungi | **716.02** | 0.796 | 718.02 | 0.965 |
| Diversity of EcM fungi | 517.17 | <0.001 | **508.23** | <0.001 |
| Diversity of endophytic fungi | 273.04 | 0.400 | **267.57** | 0.019 |
| Diversity of saprotrophic fungi | **528.35** | 0.024 | 528.66 | 0.035 |

n=84 samples in either roots and soils. The lower AIC values between linear and quadratic polynomial models are in bold.

**Table S4** Results of linear mixed-effects models (LMMs) for the elevation pattern of diversities of total fungi, EcM fungi, endophytic fungi and saprotrophic fungi with latitude and longitude as random effects.

|  | Linear | | | | | Quadratic | | | | |
| --- | --- | --- | --- | --- | --- | --- | --- | --- | --- | --- |
|  | AICc | *P* | β_fixed_ | R^2^_m_ | R^2^_c_ | AICc | *P* | β_fixed_ | R^2^_m_ | R^2^_c_ |
| **Roots:** |  |  |  |  |  |  |  |  |  |  |
| Diversity of total fungi | **908.1** | **0.004** | -0.12 | 0.107 | 0.388 | 909.3 | **0.007** | -172.4, -63.1 | 0.135 | 0.375 |
| Diversity of EcM fungi | 693.2 | **0.006** | -0.04 | 0.113 | 0.533 | **690.4** | **<0.001** | -54.0, -38.7 | 0.214 | 0.535 |
| Diversity of endophytic fungi | **468.5** | 0.436 | -0.00 | 0.009 | 0.133 | 469.4 | 0.358 | -3.1, 4.6 | 0.027 | 0.130 |
| Diversity of saprotrophic fungi | **714.1** | **0.030** | -0.03 | 0.063 | 0.307 | 716.3 | 0.088 | -39.0, -5.2 | 0.067 | 0.303 |
| **Soils:** |  |  |  |  |  |  |  |  |  |  |
| Diversity of total fungi | **961.2** | 0.823 | 0.01 | 0.001 | 0.012 | 963.5 | 0.973 | 15.6, -5.2 | 0.001 | 0.013 |
| Diversity of EcM fungi | 761.6 | **<0.001** | -0.07 | 0.176 | 0.412 | **753.7** | **<0.001** | -100.3, -68.0 | 0.309 | 0.309 |
| Diversity of endophytic fungi | 511.4 | 0.426 | -0.00 | 0.011 | 0.574 | **508.1** | **0.030** | -1.8, 14.5 | 0.100 | 0.524 |
| Diversity of saprotrophic fungi | **772.6** | **0.049** | 0.04 | 0.055 | 0.276 | 773.2 | **0.041** | 56.1, 34.8 | 0.093 | 0.311 |

AICc: corrected Akaike Information Criterion for small data sets; β_fixed_: Estimate of fixed effect, i.e., slope of relationships between diversities and elevation in linear models, and first-order and second-order coefficients in quadratic models; R^2^_m_: the variance explained by fixed effects; R^2^_c_: the variance explained by both fixed and random effects. Significant *P* values and the lower AIC values between linear and quadratic polynomial models are in bold. n=84 samples in either roots and soils.

**Table S5** Pearson correlations between EcM fungal diversity and 52 candidate variables in roots and soils.

|  | Roots | | Soils | |
| --- | --- | --- | --- | --- |
|  | r | *P* | r | *P* |
| Elevation | -0.38 | <0.001 | -0.46 | <0.001 |
| Slope | -0.34 | 0.002 | -0.36 | 0.001 |
| Soil moisture | -0.20 | 0.067 | -0.05 | 0.625 |
| pH | 0.02 | 0.887 | 0.08 | 0.487 |
| Conductivity | -0.49 | <0.001 | -0.49 | <0.001 |
| Nitrate nitrogen | 0.01 | 0.932 | -0.05 | 0.633 |
| Ammonium nitrogen | 0.19 | 0.082 | 0.37 | 0.001 |
| Dissolved organic nitrogen | 0.04 | 0.736 | 0.14 | 0.192 |
| Dissolved organic carbon | 0.11 | 0.343 | 0.24 | 0.026 |
| Total carbon | -0.09 | 0.407 | 0.08 | 0.489 |
| Total nitrogen | -0.03 | 0.794 | 0.16 | 0.145 |
| Clay | 0.14 | 0.195 | 0.22 | 0.04 |
| Silt | 0.26 | 0.017 | 0.28 | 0.010 |
| Sand | -0.21 | 0.052 | -0.26 | 0.016 |
| C/N ratio | -0.12 | 0.297 | -0.13 | 0.249 |
| N/P ratio | -0.09 | 0.423 | -0.16 | 0.158 |
| Total phosphate | 0.04 | 0.697 | 0.25 | 0.024 |
| Total potassium | -0.12 | 0.274 | -0.31 | 0.004 |
| Total calcium | -0.19 | 0.092 | -0.18 | 0.106 |
| Total magnesium | 0.32 | 0.003 | 0.39 | <0.001 |
| Total manganese | 0.01 | 0.933 | 0.01 | 0.899 |
| Total aluminum | 0.02 | 0.885 | -0.03 | 0.805 |
| Total iron | 0.15 | 0.172 | 0.01 | 0.931 |
| Available phosphate | -0.08 | 0.491 | 0.03 | 0.762 |
| Available potassium | 0.02 | 0.848 | 0.09 | 0.408 |
| Available calcium | -0.08 | 0.464 | -0.12 | 0.260 |
| Available magnesium | 0.12 | 0.295 | 0.00 | 0.974 |
| Available manganese | 0.09 | 0.398 | -0.02 | 0.883 |
| Available aluminum | 0.12 | 0.267 | 0.03 | 0.811 |
| Available iron | 0.20 | 0.073 | 0.07 | 0.506 |
| Root total carbon | 0.36 | 0.001 | **0.54** | **<0.001** |
| Root total nitrogen | 0.09 | 0.411 | 0.22 | 0.041 |
| Root phosphate | -0.31 | 0.004 | -0.26 | 0.018 |
| Root potassium | -0.15 | 0.177 | -0.31 | 0.004 |
| Root calcium | 0.06 | 0.570 | 0.11 | 0.343 |
| Root magnesium | -0.20 | 0.068 | -0.26 | 0.016 |
| Root manganese | 0.07 | 0.549 | -0.03 | 0.772 |
| Root aluminum | 0.05 | 0.656 | 0.12 | 0.277 |
| Root iron | 0.21 | 0.061 | 0.18 | 0.094 |
| Root C/N ratio | -0.12 | 0.287 | -0.23 | 0.037 |
| Root N/P ratio | 0.38 | <0.001 | 0.37 | <0.001 |
| Lignin | 0.37 | 0.001 | 0.44 | <0.001 |
| Cellulose | 0.32 | 0.003 | 0.53 | <0.001 |
| Hemicellulose | 0.29 | 0.007 | 0.43 | <0.001 |
| Soluble sugar | 0.31 | 0.001 | 0.46 | <0.001 |
| Soluble protein | 0.28 | 0.001 | 0.40 | <0.001 |
| Free amino acid | 0.42 | <0.001 | 0.33 | 0.003 |
| Free fatty acid | 0.40 | <0.001 | 0.49 | <0.001 |
| Tree height | **0.50** | **<0.001** | 0.46 | <0.001 |
| Canopy diameter | 0.39 | <0.001 | 0.36 | 0.001 |
| Diameter at breast height | **0.50** | **<0.001** | 0.43 | <0.001 |
| Distance to forest edge | -0.48 | <0.001 | -0.49 | <0.001 |

The strongest correlates with EcM fungal diversity in roots and soils are in bold. n=84 samples in either roots and soils.

**Table S6** Results of linear mixed-effects models (LMMs) for EcM fungal diversity-DBH, EcM fungal diversity-RTC and EcM fungal diversity-DFE relationships with latitude and longitude as random effects.

|  | *P* | β_fixed_ | R^2^_m_ | R^2^_c_ |
| --- | --- | --- | --- | --- |
| **Roots:** |  |  |  |  |
| EcM fungal diversity-DBH relationship | **<0.001** | 0.67 | 0.223 | 0.538 |
| EcM fungal diversity-RTC relationship | **0.026** | 3.81 | 0.059 | 0.491 |
| EcM fungal diversity-DFE relationship | **<0.001** | -0.10 | 0.226 | 0.496 |
| **Soils:** |  |  |  |  |
| EcM fungal diversity-DBH relationship | **<0.001** | 0.96 | 0.190 | 0.190 |
| EcM fungal diversity-RTC relationship | **<0.001** | 13.34 | 0.296 | 0.296 |
| EcM fungal diversity-DFE relationship | **<0.001** | -0.16 | 0.239 | 0.243 |

β_fixed_: Estimate of fixed effect, i.e., estimates of species-area, species-energy and species-isolation relationships; R^2^_m_: the variance explained by fixed effects; R^2^_c_: the variance explained by both fixed and random effects. Significant *P* values are in bold. n=84 samples in either roots and soils. Here, diameter at breast height (DBH), root total carbon (RTC) and distance to forest edge (DFE) represent the area, energy and isolation of “virtual islands”, respectively, based on the island biogeography theory (IBT) framework.

**Table S7** Summary of the best ordinary least squares (OLS) multiple regression models for the effects of environmental variables, elevation gradient and biotic interactions on saprotrophic fungal diversity in roots and soils.

| Predictors | Estimate | *t*-value | *P*-value | VIF | R^2^_adj.cum_ |
| --- | --- | --- | --- | --- | --- |
| **Roots:** df=81, R^2^_adj_=0.508, SE_resid_=0.702, *P*<0.001, AIC=-56.55 | | | | | |
| Diversity of EcM fungi in roots | 0.53 | 6.87 | <0.001 | 1.01 | 0.313 |
| Diversity of endophytic fungi in roots | 0.45 | 5.78 | <0.001 | 1.01 | 0.508 |
| **Soils:** df=74, R^2^_adj_=0.860, SE_resid_=0.374, *P*<0.001, AIC=-155.87 | | | | | |
| Diversity of endophytic fungi in soils | 0.61 | 12.07 | <0.001 | 1.50 | 0.618 |
| DBH | -2.16 | -3.61 | <0.001 | 2.12 | 0.684 |
| pH | 0.23 | 5.16 | <0.001 | 1.15 | 0.724 |
| Slope | 0.16 | 3.16 | 0.002 | 1.44 | 0.757 |
| Diversity of EcM fungi in soils | 0.30 | 5.59 | <0.001 | 1.76 | 0.806 |
| Conductivity | 0.23 | 3.83 | <0.001 | 2.10 | 0.828 |
| PCNM2 | -0.14 | -2.64 | 0.010 | 1.73 | 0.842 |
| Total potassium | -0.17 | -3.25 | 0.002 | 1.65 | 0.852 |
| Silt | -0.13 | -2.31 | 0.024 | 1.90 | 0.860 |

AIC, Akaike’s information criterion; VIF, variance inflation factor. n=84 samples in either roots and soils. Notably, PCNM1-5, as the proxies of geographic effects, are also added in the models.

**Table S8** Summary of the best ordinary least squares (OLS) multiple regression models for the effects of environmental variables, elevation gradient and biotic interactions on endophytic fungal diversity in roots and soils.

| Predictors | Estimate | *t*-value | *P*-value | VIF | R^2^_adj.cum_ |
| --- | --- | --- | --- | --- | --- |
| **Roots:** df=81, R^2^_adj_=0.325, SE_resid_=0.822, *P*<0.001, AIC=-30.02 | | | | | |
| Diversity of saprotrophic fungi in roots | 0.56 | 6.07 | <0.001 | 1.05 | 0.230 |
| DBH | -0.33 | -3.53 | <0.001 | 1.05 | 0.325 |
| **Soils:** df=79, R^2^_adj_=0.762, SE_resid_=0.488, *P*<0.001, AIC=-115.75 | | | | | |
| Diversity of saprotrophic fungi in soils | 0.84 | 15.58 | <0.001 | 1.02 | 0.618 |
| Soluble protein | 0.28 | 4.70 | <0.001 | 1.24 | 0.718 |
| PCNM2 | 0.08 | 4.06 | <0.001 | 1.70 | 0.741 |
| Root total potassium | 0.19 | 2.84 | 0.006 | 1.63 | 0.762 |

AIC, Akaike’s information criterion; VIF, variance inflation factor. n=84 samples in either roots and soils. Notably, PCNM1-5, as the proxies of geographic effects, are also added in the models.

**
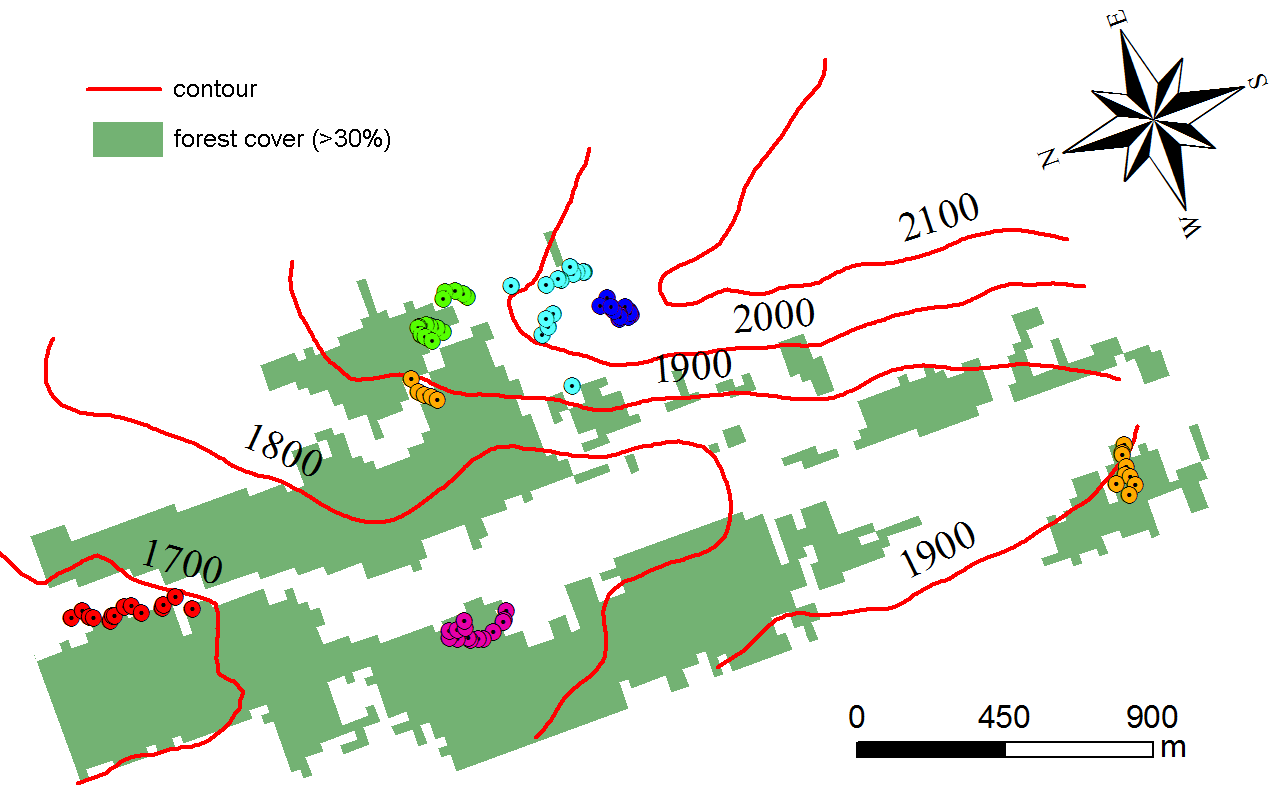
Fig. S1** The sampling map of *B. ermanii* along the timberline in Changbai Mountain. The data is from treecover2000 with 30-m resolution (https://storage.googleapis.com/earthenginepartners-hansen/GFC-2017-v.1.5/Hansen_GFC-2017-v1.5_treecover2000_40N_080W.tif), and implemented in ArcGIS 10.0 (ESRI, Redlands, CA, USA). The areas where data values are more than 30 are shown. In the island biogeography theory (IBT) framework, the green grids are defined as the “mainland”, and the minimum distance of each sampling point to the green grids were calculated as distance to forest edge (DFE).

**
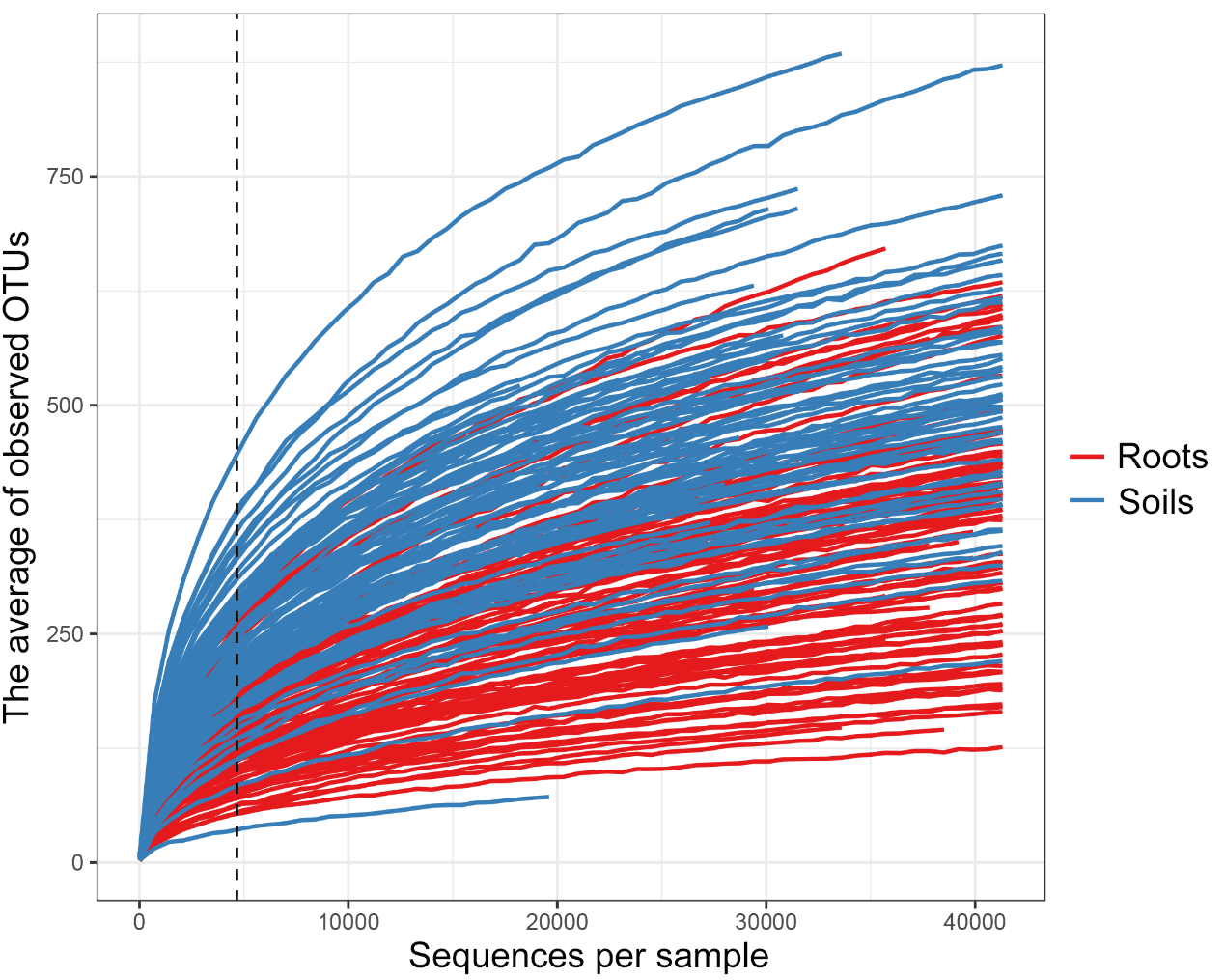
Fig. S2** The rarefaction curves of the average observed OTU number in roots and soils. The vertical black line represent the minimum sequence number (4662) per sample.

**
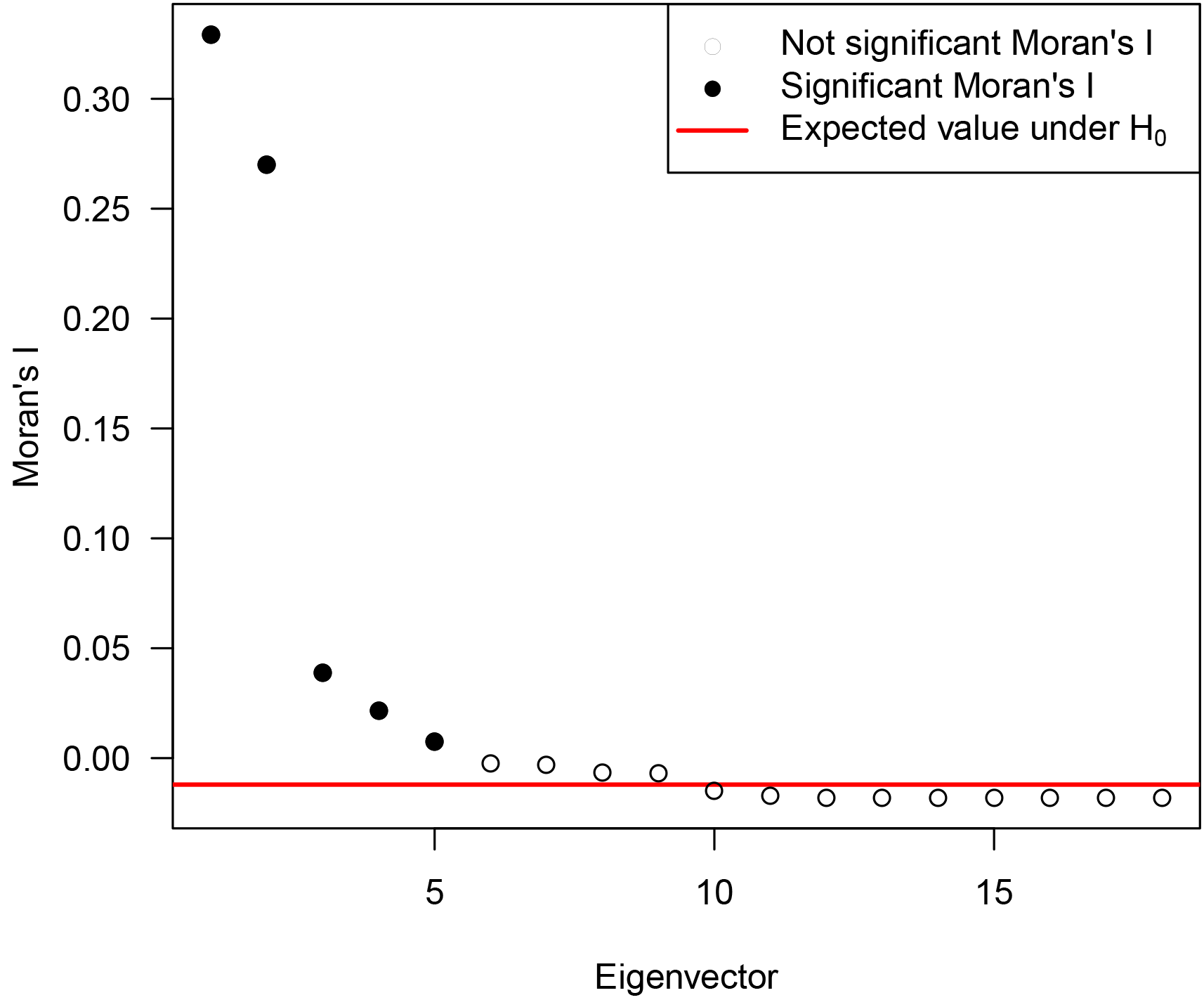
Fig. S3** The plot of automatic construction of the spatial PCNM variables and calculation of the Moran’s I for each variable.


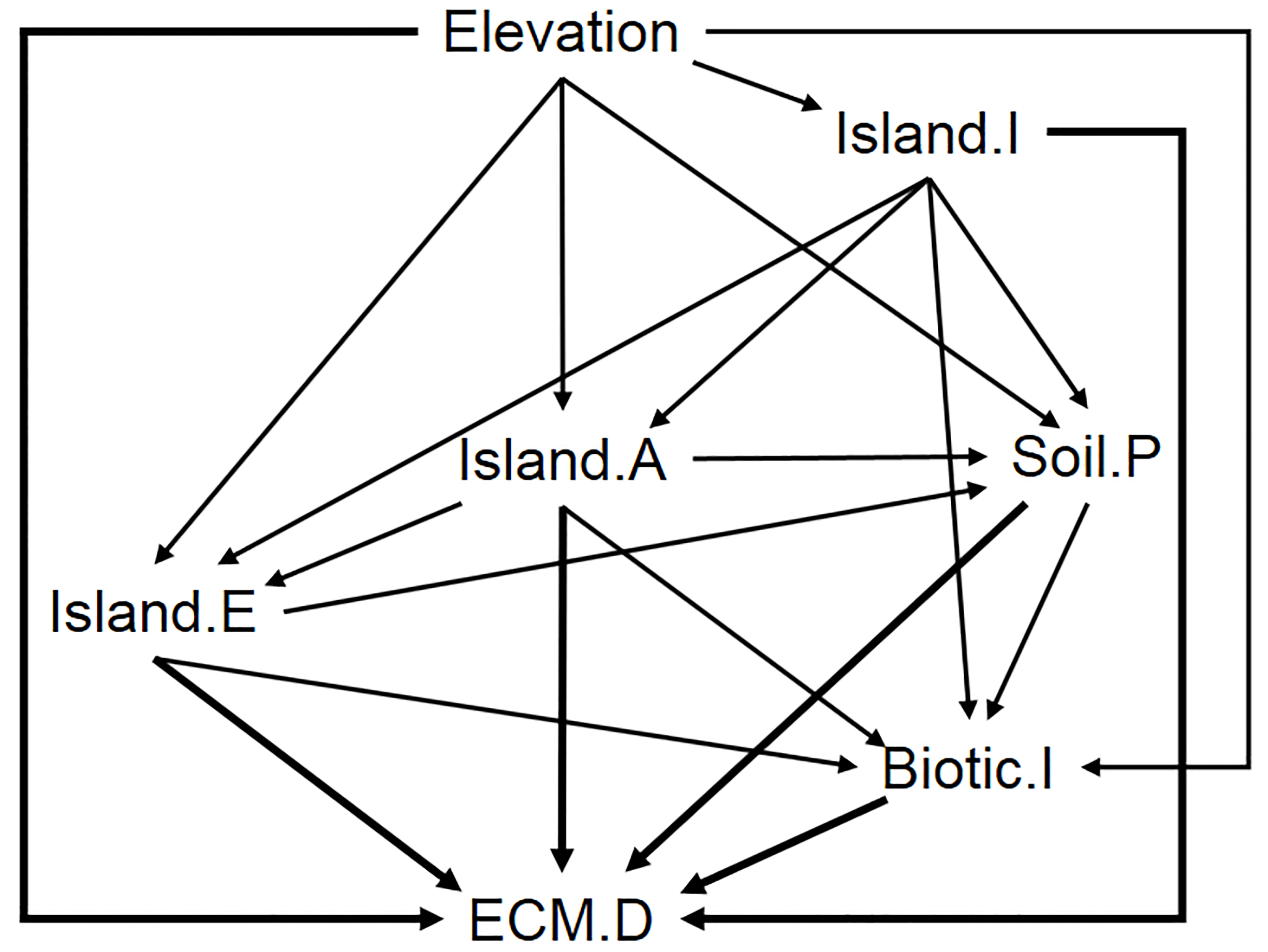
**Fig. S4** SEM theoretical model in the present study. The paths of direct effects on EcM fungal diversities are in bold. Island.I: island isolation, Island.A: island area, Island.E: island energy, Soil.P: soil properties, Biotic.I: biotic interactions, ECM.D: diversity of EcM fungi. Here, the seven labels except ECM.D are treated as ‘predictive aspects’. In sum, SEM theoretical model mainly comprises the IBT framework (i.e., the island area, energy and isolation effects), followed by the climate-driven hypothesis with the elevation gradient, biotic interactions (i.e., the saprotrophic diversity effect) and the edaphic effect.

**
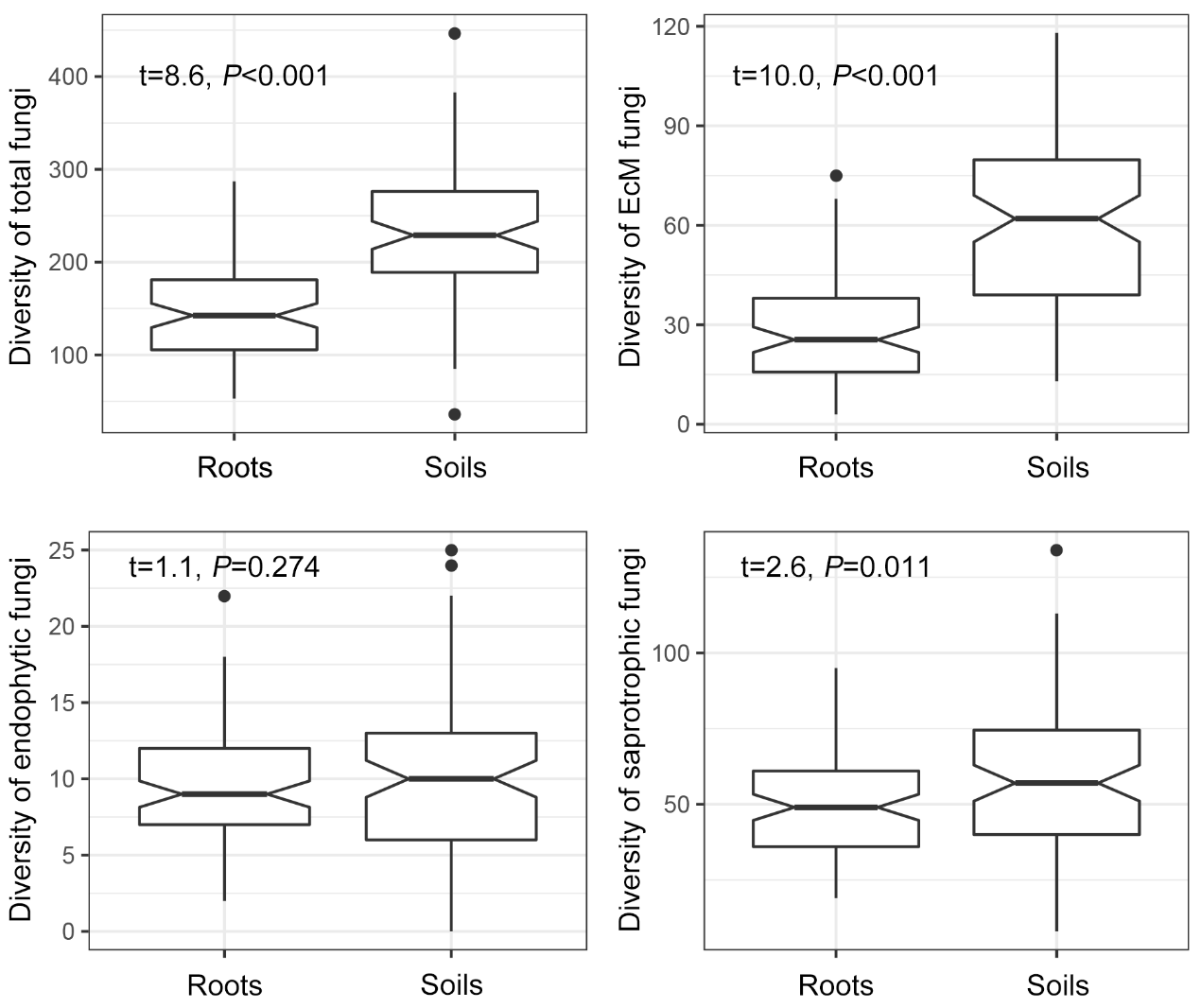
Fig. S5** Comparison of fungal diversity (total fungi, EcM fungi, endophytic fungi and saprotrophic fungi) between roots and soils. In the diagram, t and *P* values of independent t tests are shown. n=84 in roots and soils, respectively.

**
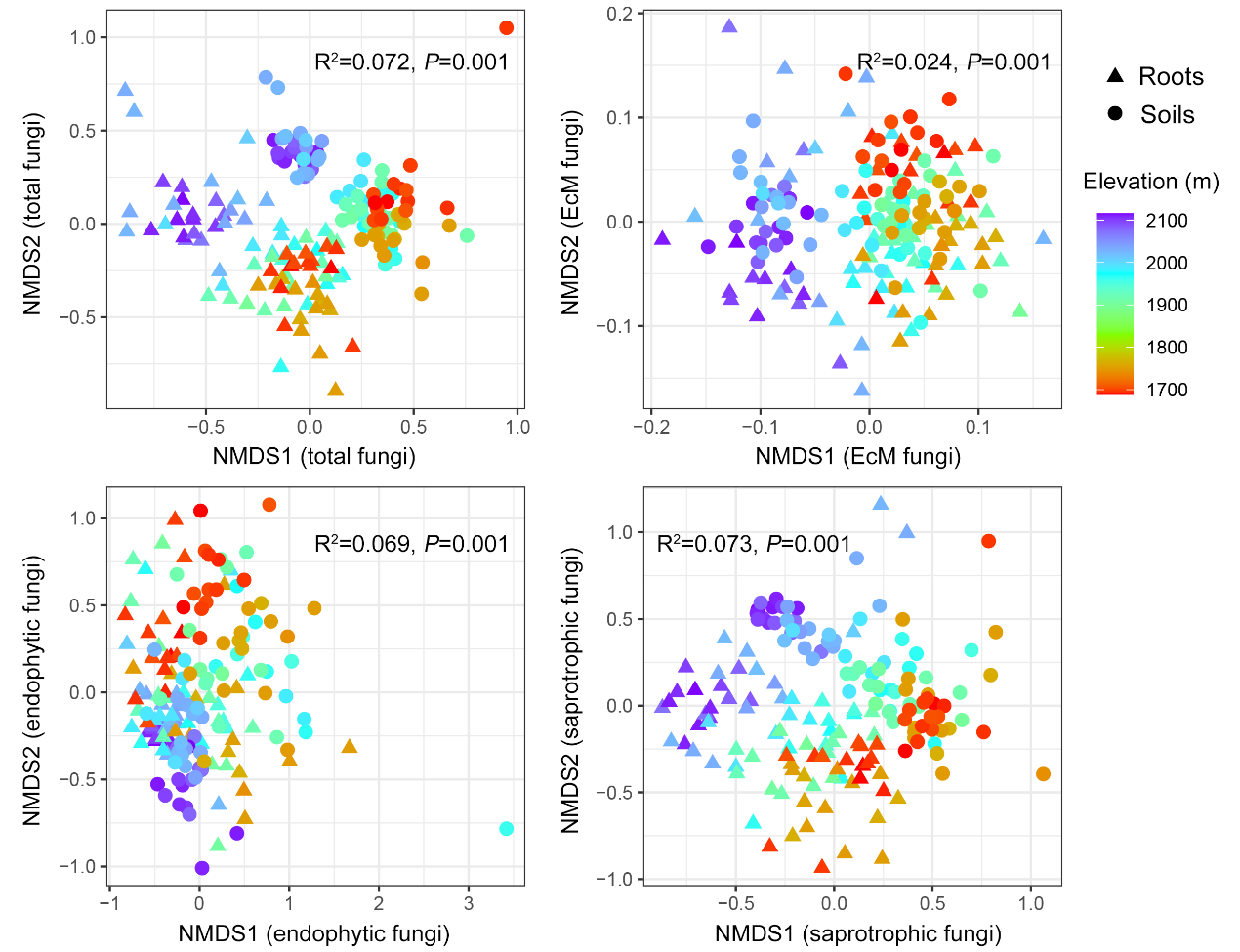
Fig. S6** Two-dimensional ordination of the variation in fungal community composition (total fungi, EcM fungi, endophytic fungi and saprotrophic fungi) using NMDS, based on Bray-Curtis dissimilarity matrix. Roots and soils are represented by different shapes. Elevation gradient is shown in a color bar. The significant difference in community composition between roots and soils is tested by PERMANOVA, and is shown in the diagram. In addition, there is the significant variation in community composition along the elevational gradient (total fungi: R^2^=0.058 *P*=0.001; EcM fungi: R^2^=0.044, *P*=0.001; endophytic fungi: R^2^=0.070, *P*=0.001; saprotrophic fungi: R^2^=0.075, *P*=0.001). n=84 in roots and soils, respectively.

**
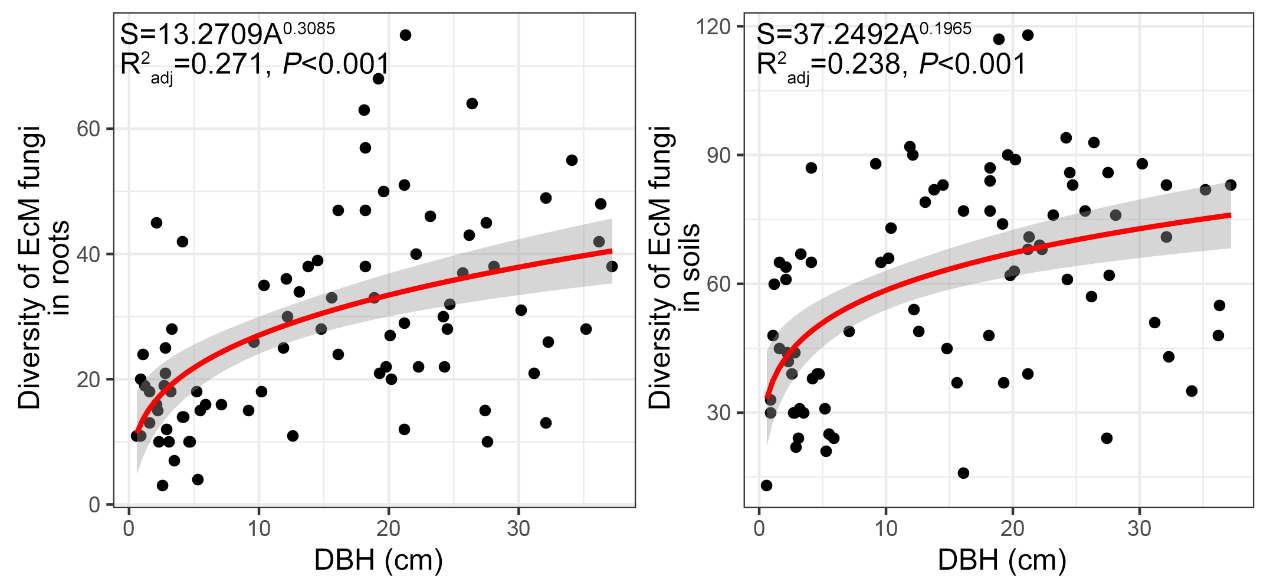
Fig. S7** The area-species curve of EcM fungi in roots and soils. Here, the equation S=CA^z^ was shown in diagram. S is the number of EcM fungal OTUs, and A is DBH representing the area of ‘virtual island’. Adjusted R squares and *P* values of power law functions are shown. n=84 samples in either roots and soils.

**
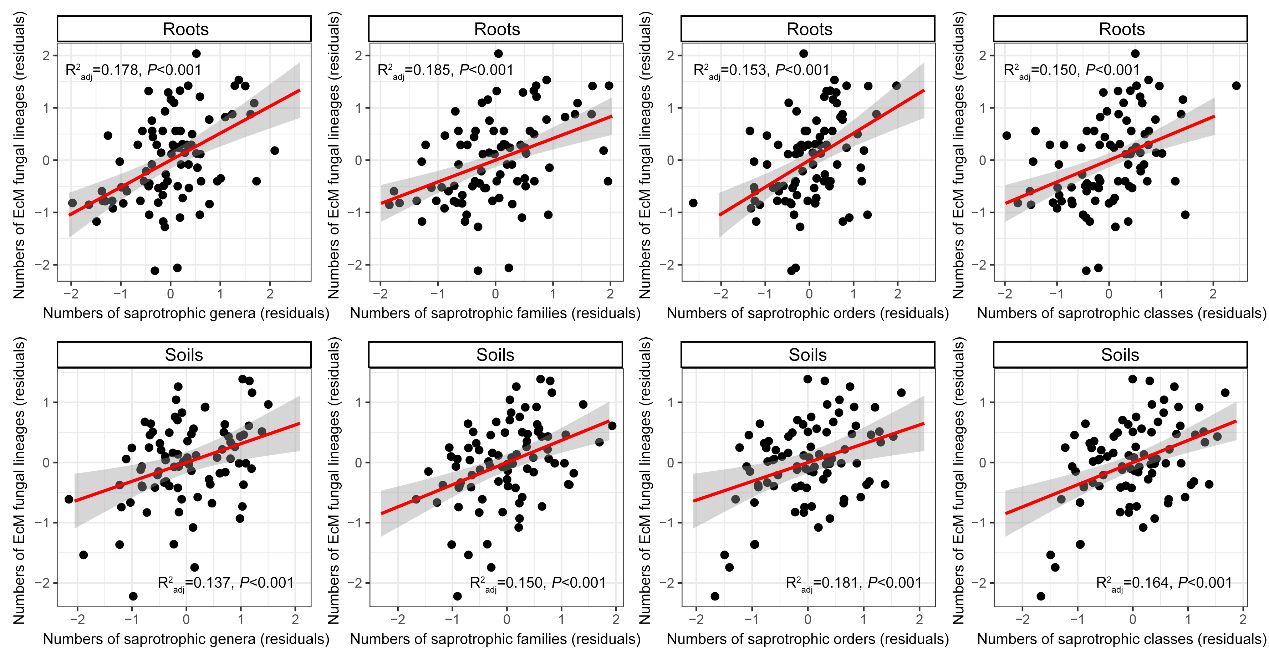
Fig. S8** Relationships between numbers of EcM fungal lineages and saprotrophic richness at the genus, family, order and classes levels, when accounting for multiple predictors. The residues of numbers of EcM fungal lineages and saprotrophic fungal richness are fitted by partial least squares regression (PLSR). The solid red lines indicate statistical significance for the relationships, and the shaded areas show the 95% confidence interval of the fit. n=84 samples in either roots and soils.


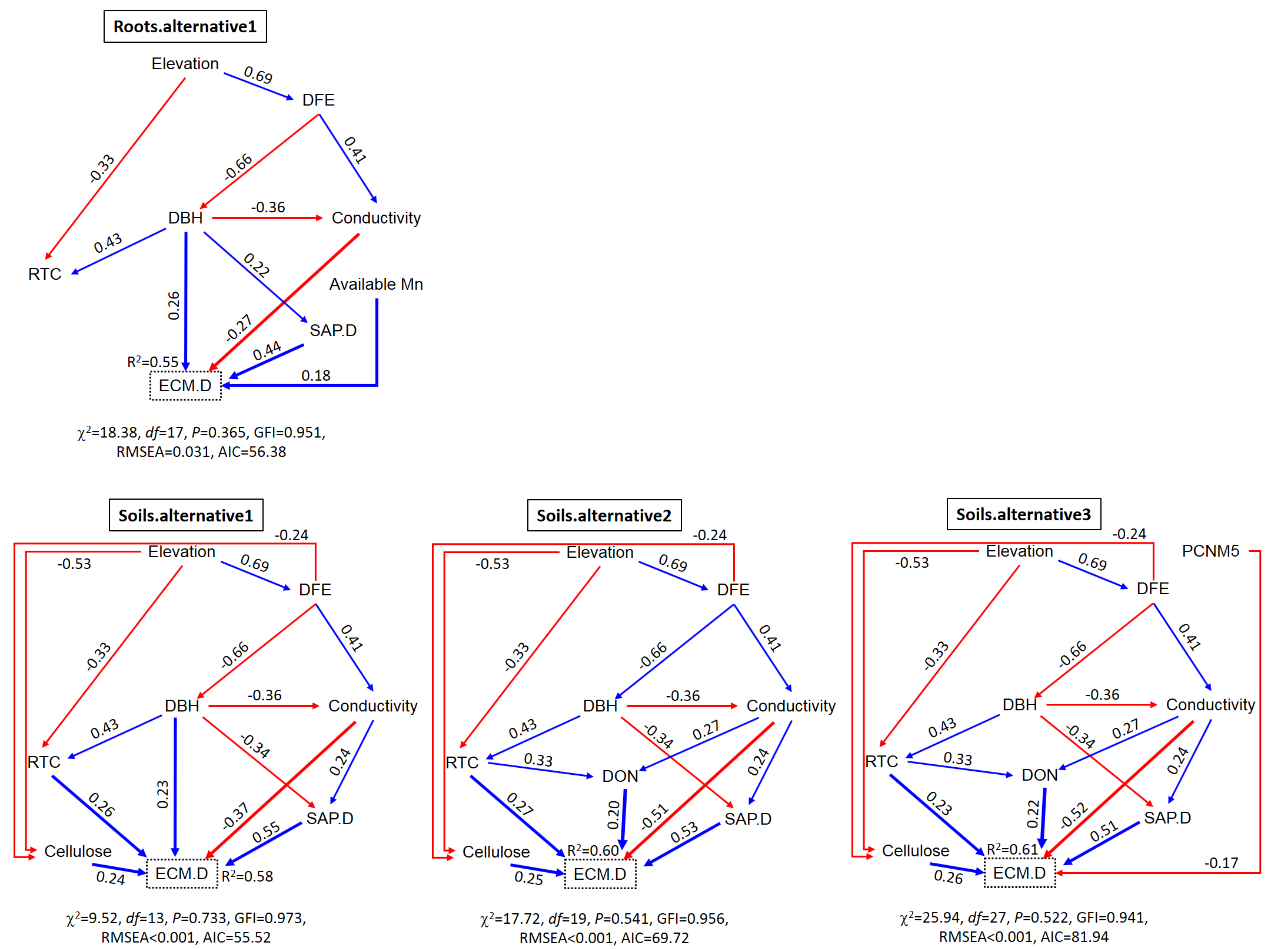
**Fig. S9** The alternative SEM models for EcM fungal diversity in roots and soils. The paths of direct effects on EcM fungal diversity are in bold, and only significant paths are retained. Blue color indicates the positive effect, while red color indicates the negative effect. Standardized path coefficient (SPC) is shown near each corresponding path. GFI: goodness of fit index, RMSEA: root mean square error of approximation, SAP.D: saprotrophic fungal diversity, ECM.D: EcM fungal diversity. n=84 samples in either roots and soils.

**
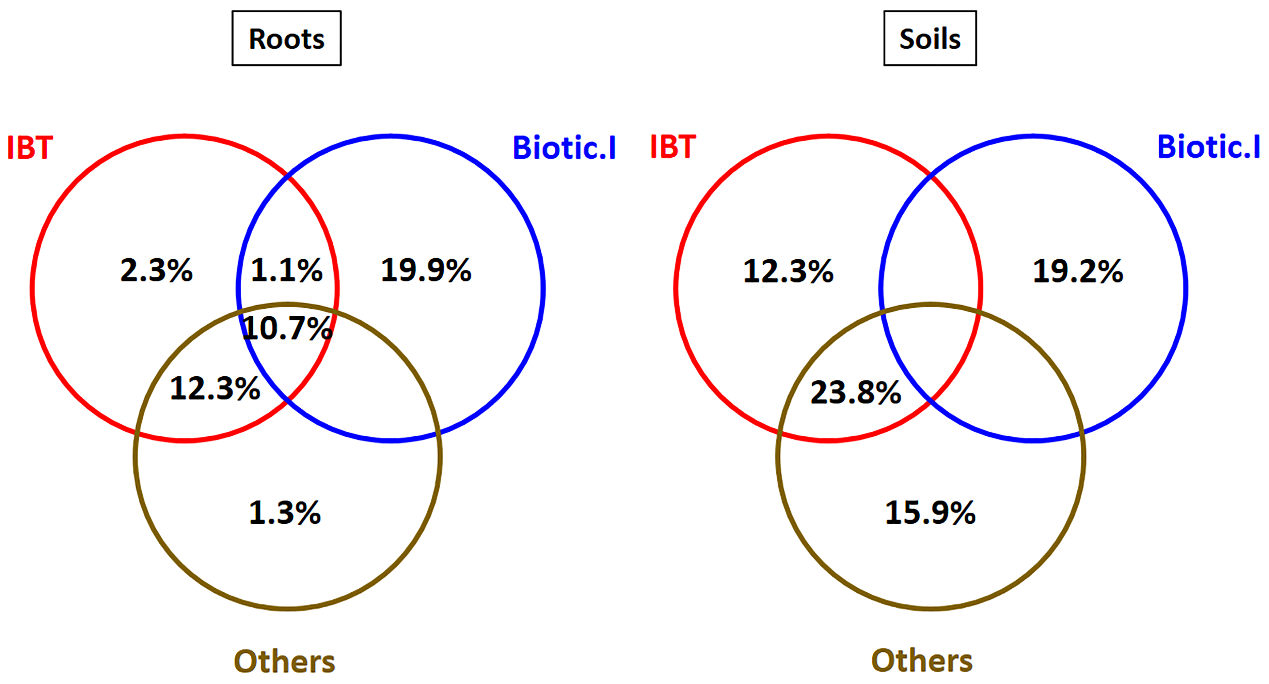
Fig. S10** A Venn diagram of variation partitioning analysis, illustrating the shared and exclusive effects of IBT-involved elements, biotic interactions and other predictors on EcM fungal diversity in roots and soils. The fraction of unexplained variation and values < 0.1% are not shown for simplicity. Here, DBH, RTC and DFE are IBT-invovled elements, and saprotrophic fungal diversity represents biotic interactions. Other predictors include elevation and soil properties. n=84 samples in either roots and soils.

**
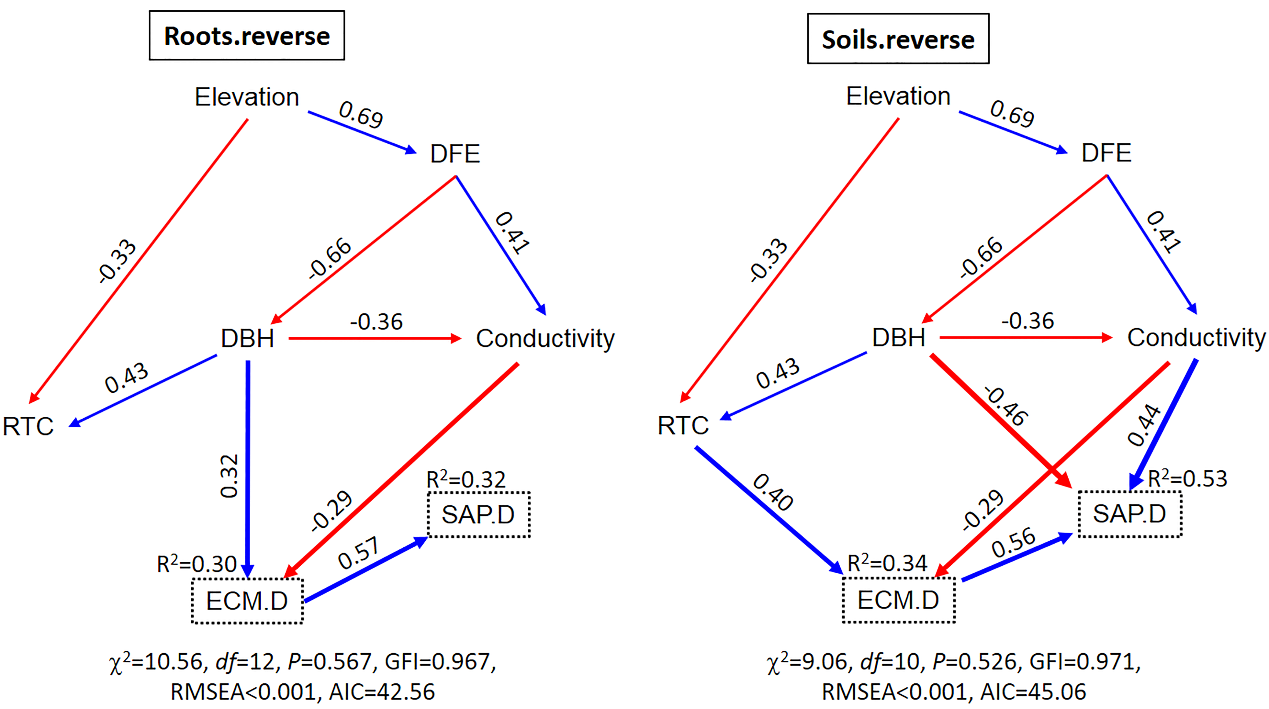
Fig. S11** The reverse SEM models that assume EcM fungal diversity directly affects saprotrophic fungal diversity. The paths of direct effects on EcM and saprotrophic fungal diversities are in bold, and only significant paths are retained. Blue color indicates the positive effect, while red color indicates the negative effect. SPC is shown near each corresponding path. GFI: goodness of fit index, RMSEA: root mean square error of approximation, SAP.D: saprotrophic fungal diversity, ECM.D: EcM fungal diversity. n=84 samples in either roots and soils.
